# Structural basis for ADAM17 activation by the iRhom1 pseudoprotease

**DOI:** 10.1016/j.celrep.2026.117309

**Published:** 2026-04-22

**Authors:** Joseph N. Ungvary, Joseph J. Maciag, Hala F. Alnajjar, Eliud Morales Dávila, Conner E. Slone, Joel E. Thomas, Maria F. Rich, Sophia A. Carazo, Igal Ifergan, Jose Manuel Perez-Aguilar, Carl P. Blobel, Tom C.M. Seegar

**Affiliations:** 1 Department of Molecular and Cellular Biosciences, University of Cincinnati College of Medicine, Cincinnati, OH 45267, USA; 2 School of Chemical Sciences, Meritorious Autonomous University of Puebla, University City, Puebla 72570, Mexico; 3 Department of Medicine and of Biochemistry, Cell and Molecular Biology, Weill Cornell Medicine, New York, NY 10021, USA; 4 Inflammation and Autoimmunity Program, Hospital for Special Surgery, New York, NY 10021, USA; 5 These authors contributed equally; 6 Lead contact

## Abstract

The a disintegrin and metalloproteinase (ADAM)-17 releases pro-inflammatory cytokines, including tumor necrosis factor-α, and several epidermal growth factor receptor ligands from cells. ADAM17 is post-translationally regulated by the inactive rhomboid pseudoproteases iRhom1 and iRhom2, and dysregulation of this signaling axis contributes to diseases ranging from chronic inflammation to cancer. Here, we present the cryo-electron microscopy structure of the ADAM17 zymogen bound to iRhom1, revealing structural features essential for complex formation and protease activation. We identify a transmembrane α-helix and conserved cytoplasmic element in iRhom1, termed the re-entry loop, that functions as a molecular relay transmitting intracellular signals across the membrane to activate ADAM17. We also demonstrate that a human disease-associated iRhom1 mutation linked to cardiomyopathy disrupts ADAM17 maturation and trafficking. Finally, we apply all-atom molecular dynamics simulations to model the mature and active ADAM17-iRhom1 complex. These findings provide mechanistic insights into how iRhom1 regulates ADAM17 function across the membrane.

## In brief

Ungvary et al. present the cryo-EM structure of the iRhom1 pseudoprotease bound to the ADAM17 zymogen. Structural and functional studies reveal a conserved signaling axis spanning the iRhom cytoplasmic and membrane regions that transmits intracellular signals to control ADAM17 activation and substrate processing at the cell surface.

## Graphical Abstract

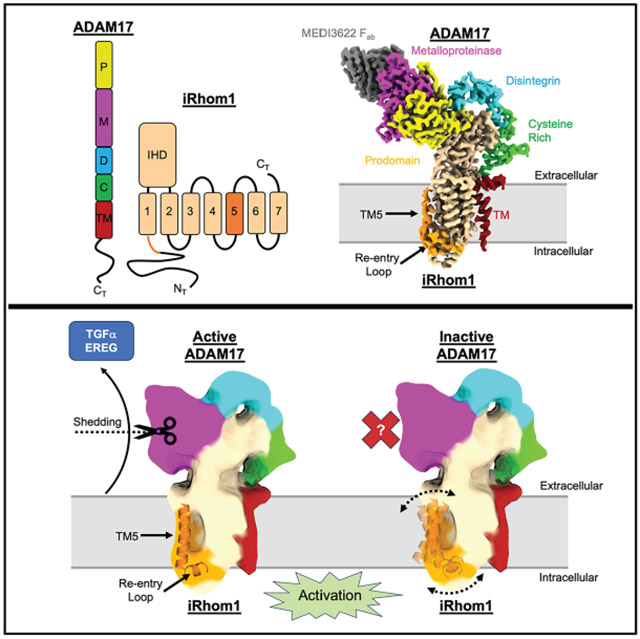

## INTRODUCTION

Ectodomain shedding, the process by which membrane-anchored proteins are proteolytically cleaved and released from the cell membrane, is essential for intercellular communication and cellular adaptation to external signals.^1^ Central to this tightly regulated process is the a disintegrin and metalloproteinase (ADAM) family of proteins, which regulate key physiological processes, including inflammation, tissue development, and cell signaling. Dysregulated ADAM activity has been implicated in a wide spectrum of diseases such as chronic inflammation, cancer, and neurodegenerative disorders.^1-5^

Among the ADAM proteases, ADAM17 (also known as tumor necrosis factor α-converting enzyme, TACE) is particularly prominent for its essential roles in epidermal growth factor receptor (EGFR) and cytokine signaling.^2,6-9^ Genetic ablation of ADAM17 in mice results in perinatal lethality and severe developmental defects, including open eyes at birth, abnormal heart valves, and impaired growth plate formation.^2,6,7,9-11^ These defects are a consequence of decreased EGFR signaling due to a loss of EGFR ligand cleavage from the cell surface.^12^ In humans, loss of ADAM17 function results in severe skin and intestinal barrier defects, likely due to impaired EGFR signaling, emphasizing its critical role in maintaining tissue integrity and homoeostasis.^13-16^ In addition to its role in development, ADAM17 also regulates immune responses by processing many immunomodulatory substrates, including tumor necrosis factor alpha (TNFα),^7,17,18^ interleukin-6 receptor (IL-6R),^19^ and tumor necrosis factor receptors 1 and 2 (TNFR1 and TNFR2).^20^ The central role of ADAM17 in immune cell function makes it a compelling pharmacological target in chronic inflammatory diseases.^5^

ADAM17 is a single-pass transmembrane protein synthesized as an inactive zymogen in the endoplasmic reticulum (ER), where its prodomain prevents premature proteolytic activity.^21^ The prodomain is removed by proprotein convertases during transit through the secretory pathway system, a step considered a pre-requisite for the activation of ADAM17. The mature ADAM17 on the cell surface contains an extracellular metalloproteinase (M), disintegrin (D), and cysteine-rich (C) domain, a single transmembrane (TM) α-helix, and a cytoplasmic domain. Proteolytic shedding of ADAM17 substrates can be rapidly induced with a variety of stimuli, including the activation of protein kinases and G protein-coupled receptors (GPCRs).^12,22-25^ While the cytoplasmic region of ADAM17 is required for normal embryonic development in mice,^26^ only the transmembrane and ectodomains are essential for regulating stimulus-induced shedding of protein substrates in cells.^22,27^ The finding that the cytoplasmic domain of ADAM17 is dispensable for its rapid activation by intracellular signaling cascades is perplexing and raises important questions about how ADAM17 senses and integrates these signals. As a result, the molecular mechanisms that regulate ADAM17 activity on the cell surface remain incompletely understood.

The discovery of the inactive rhomboid proteins (iRhom1 and iRhom2) as key regulators of ADAM17 maturation and function has fundamentally advanced our understanding of ADAM17 regulation.^28-32^ Loss of both iRhom1 and iRhom2 in mice causes perinatal lethality with developmental phenotypes nearly identical to those observed in ADAM17-deficient mice, including open eyes at birth and defective cardiac valve morphogenesis, underscoring the indispensable role of iRhoms in regulating ADAM17 function.^31^ iRhom1 and iRhom2 share approximately 61% sequence identity, yet their individual ablation in mice results in non-overlapping manifestations.^28,29,31-33^ Mice lacking iRhom2, which have no spontaneous pathological phenotypes, fail to generate mature, functional ADAM17 in hematopoietic cells, leading to strongly reduced TNFα secretion under proinflammatory conditions^33^ and protection from TNFα-dependent pathologies such as endotoxin shock, inflammatory arthritis, and hemophilia arthropathy.^28,33,34^ In contrast, iRhom1-deficient mice also appear normal, even though they lack mature ADAM17 in the brain (except in microglia).^31^ A second *iRhom1*^−/−^ mouse strain, generated with a different gene targeting strategy, suffers from neurological defects and increased mortality, suggesting tissue-specific functions.^32^ Cell-based studies have further revealed that iRhom paralogs exhibit distinct substrate selectivity, in that iRhom2 preferentially supports the stimulated shedding of substrates such as HB-EGF or epiregulin (EREG), whereas both iRhom1 and iRhom2 can control the release of the EGFR ligand TGFα,^35^ which is essential for protecting the skin and intestinal barrier.^8^

iRhoms are members of the rhomboid-like superfamily of intramembrane proteases that have evolved into catalytically inactive pseudoproteases, lacking the serine-histidine dyad required for proteolysis.^36,37^ In addition, these proteins possess an extended cytoplasmic region and a large extracellular domain (ECD) known as the iRhom homology domain (IHD). Recent cryo-EM structures have revealed that iRhom2 binds directly to the zymogen ADAM17 through an extended interface across the extracellular and transmembrane regions, which serves to tether ADAM17 and facilitate its exit from the ER and processing of its protein substrates.^38,39^ Although the extracellular and transmembrane interactions are critical for complex formation and enzyme activity,^22,40,41^ the cytoplasmic domains of iRhoms are essential for regulating stimulated ADAM17 activity.^42-46^ These regions harbor multiple phosphorylation sites and adaptor protein-binding motifs that are hypothesized to relay intracellular signaling events, such as those initiated by MAPK cascades or GPCRs, to conformational rearrangements in the membrane-embedded complex. However, the molecular mechanisms by which cytoplasmic cues modulate ADAM17 activation remain poorly defined. Importantly, structural and functional characterization of iRhom1, which shares the same core architecture but has distinct substrate selectivity compared to iRhom2, is lacking and represents a critical gap in our understanding of ADAM17 regulation.

In this study, we present the cryo-EM structure of iRhom1 bound to zymogen ADAM17, together with a structure-function analysis, revealing the molecular basis for ADAM17 regulation by iRhom1. Our findings show how iRhom1 engages ADAM17 and highlight the essential role of the conserved re-entry loop within the iRhom1 cytoplasmic domain in supporting ADAM17 proteolytic function. In addition, we characterize the iRhom1 TM5 and re-entry loop, which do not directly contact ADAM17, as critical structural elements that modulate substrate processing. Finally, we present molecular dynamics (MD) simulations that place the metalloproteinase domain of mature ADAM17 above these regulatory helices, suggesting a mechanism by which iRhom1 positions ADAM17 to promote efficient substrate cleavage. These results provide key mechanistic insights into the ADAM17 interaction with both iRhom1 and iRhom2, offering a structural framework for therapeutic strategies targeting dysregulated ADAM17 activity in disease.

## RESULTS

### Structure of zymogen ADAM17-iRhom1 complex

**Figure 1.**
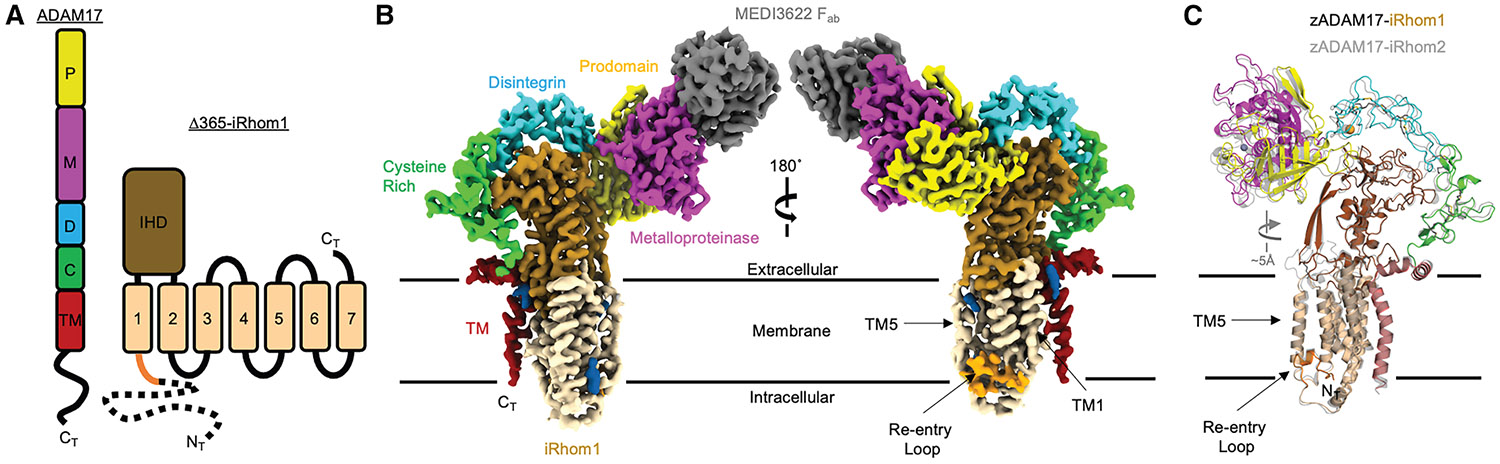
Overall structure of the zymogen ADAM17-iRhom1 complex (A) Schematic of ADAM17 colored by domain: pro-domain (P, yellow), metalloproteinase (M, magenta), disintegrin (D, cyan), cysteine-rich (C, green), and transmembrane helix (TM, red). The 7-pass transmembrane Δ365-iRhom1 (tan) with the individual TM numbers 1–7 and the iRhom homology domain (brown) between TM1 and TM2 are indicated. The re-entry loop is colored orange, and the portion of the iRhom1 N_T_ removed from this structure is indicated with dotted lines. (B) Cryo-EM map of the zymogen ADAM17-iRhom1-MEDI3622 F_ab_ complex within the cell membrane, which is indicated by parallel black lines. The maps are colored based on the domain organization, schematically represented in (A), with the addition of the MEDI3622 F_ab_ and putative lipid molecules, which are colored in gray and blue, respectively. (C) Cartoon representation and superimposition of the zymogen ADAM17-iRhom1 complex (in color) and zymogen ADAM17-iRhom2 complex (gray, PDB: 9O58).^39^ The iRhom TM5 and re-entry loop are indicated with arrows. The ADAM17-bound calcium and catalytic zinc ions are represented as spheres. The gray arrow indicates the interdomain movement, ~5 Å, of the pro-M domain of ADAM17 bound to iRhom2 compared to iRhom1.

iRhoms are multi-pass transmembrane proteins with an extensive cytoplasmic region that is predicted to be largely unstructured and intrinsically flexible, posing challenges for biochemical stability and structural analysis. In our previous work, we found that full-length iRhom2 was highly susceptible to proteolysis during purification and therefore developed an expression construct lacking much of the cytoplasmic region, while another study required the co-expression of the iRhom cytoplasmic binding protein FRMD8 to obtain sufficient material for structure determination.^38,39^ Notably, comparison of the iRhom2 structures generated with the majority of the cytoplasmic region co-expressed with FRMD8 or without the majority of the cytoplasmic region revealed nearly identical architectures, indicating that truncation of this region does not alter the core iRhom fold or its interaction with ADAM17, or that binding of FRMD8 has a similar effect as deletion of the majority of the cytoplasmic domain, including its binding sequence. We therefore adopted our previously established iRhom2 expression and purification strategy and applied this approach to the generation of an ADAM17-iRhom1 complex for structural determination. Specifically, we engineered an iRhom1 expression construct in which the first 365 amino acids were replaced with mVenus (mVenus-Δ365-iRhom1). This construct was co-expressed with an inactive form of ADAM17 (E406A), purified to homogeneity in a glycol-diosgenin (GDN) micelle, and bound by an ADAM17 selective MEDI3622 F_ab_, which served as a fiducial marker. The zymogen ADAM17-iRhom1-F_ab_ complex was imaged by cryo-EM, and single-particle analysis yielded a molecular reconstruction at an overall resolution of 3.37 Å (Figures 1 and S1; Table S1). Structural models for the MEDI3622 F_ab_ and zymogen ADAM17 (PDB: 9O58), along with a predictive AlphaFold3 (AF3) model for Δ365-iRhom1, were fit into the cryo-EM map, refined, and manually adjusted to optimize alignment (Table S1). The AF3 predictive model closely matched the experimentally derived Δ365-iR-hom1 structure and readily superimposed with an overall root mean square difference (RMSD) of 1.04 Å (Figure S2). Inspection of the final protein model in the cryo-EM map revealed good correlation between the Cα-backbone and placement of large amino acid side chains into map features extending from secondary structural elements (Figure S3). However, the cytoplasmic region of ADAM17 and the mVenus fused to iRhom1 at residue 366 were not resolved in the final map, most likely due to their intrinsic flexibility, and were therefore excluded from the final model. In addition, the carboxyl-terminal region, extending from the iRhom1 TM7 into the extracellular space, is also not resolved in the final cryo-EM map.

The overall architecture of the zymogen ADAM17-iRhom1 complex reveals an extended conformation of the ADAM17 ECD, with the MEDI3622 F_ab_ bound to the M domain and projecting outward from the complex (Figure 1). The ADAM17 ECD is positioned laterally to the membrane, with its prodomain and ancillary D and C domains forming extensive contacts with the iRhom1 homology domain (HD). The iRhom HD is a distinct 240 amino acid region located between TM1 and TM2 that extends ~45 Å from the cell membrane, stabilized by a network of eight disulfide bonds, and is capped by an unstructured segment that secures the ADAM17 ECD. Both ADAM17 and iRhom1 are embedded into the membrane via their transmembrane (TM) α–helices. The TM of ADAM17 is found nestled between iRhom1 TM1 and TM2. The TM1-6 helices of iRhom1 form a rhomboid-like fold, with TM7 located peripherally to the core helical bundle.

iRhom1 and iRhom2 share 69% sequence identity within the extracellular and transmembrane domains. The cryo-EM structures of iRhom2 (PDB: 9O58 and 8SNL) are overall conserved in architecture and readily superimpose onto iRhom1 with an RMSD of 0.8 Å, indicating a high degree of structural conservation.^38,39^ Examination of both complexes reveals that iRhom1 and iRhom2 engage the zymogen ADAM17 in an almost identical manner, with the ADAM17 ectodomain draped across the iRhom HD and forming extensive contacts at the D and C domains (Figure 1C). The most notable difference between the two complexes is a subtle ~5 Å shift in the positioning of the ADAM17 pro-M and ancillary C and D domains relative to the bound iRhom protein, which may reflect slight differences in interdomain flexibility or interface stabilization.

### Function of the iRhom1 cytoplasmic region in ADAM17 activity

**Figure 2.**
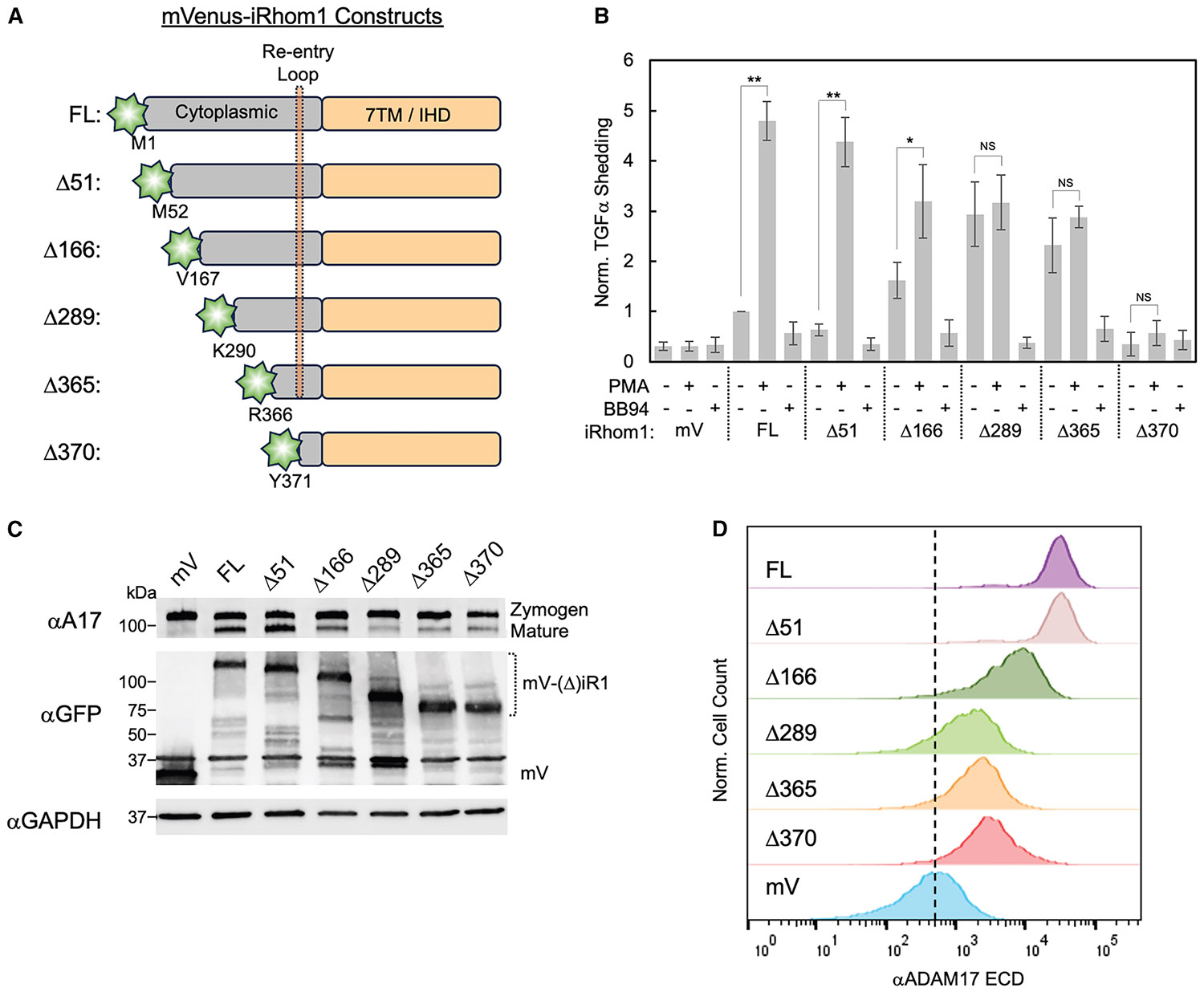
Function of the iRhom1 cytoplasmic region in ADAM17 shedding activity (A) Schematic of the chimeric mVenus-iRhom1 cytoplasmic truncations, with mVenus depicted as a green star, iRhom1 shown with the cytoplasmic region in gray, and TM1-7 region in tan. The re-entry loop is highlighted as an orange box. Deletion points and the first retained iRhom1 amino acid are noted. (B) ADAM17 shedding assays for TGFα after BB94 washout in iRhom1/2-null cells co-transfected with AP-TGFα and either mVenus (mV), mVenus-iRhom1 (FL), or mVenus-iRhom1 constructs with cytoplasmic truncations. TGFα shedding was measured under basal shedding (untreated cells) or after the addition of either PMA or BB94. Data was normalized to the basal FL-iRhom1, and statistical significance was determined using an unpaired, two-tailed *t* test (where *p < 0.05; **p < 0.005; NS, not significant). Data are represented as the mean ± SD of *N* = 3 independent experiments. (C) Western blot analysis of ADAM17 (top) in iRhom1/2-null cells transfected with either mVenus (mV), mVenus-iRhom1 (FL), or iRhom1 constructs with cytoplasmic truncations (middle, αGFP). Zymogen and mature ADAM17 bands are indicated. αGAPDH (bottom) was used as a cell lysate loading control. (D) Flow cytometry analysis of ADAM17 surface levels of iRhom1/2-null cells transfected with the mVenus-iRhom1 constructs from (A) to (C).

Previously, we reported a link between the enzymatic activity of ADAM17 and a conserved structural element in the iRhom2 cytoplasmic region, termed the “re-entry loop,” which is wedged at the base of the rhomboid fold between TM2 and TM5. In our zymogen ADAM17-iRhom1 structure, we observe that this conserved feature is similarly positioned between the iRhom1 TM2 and TM5 (Figure 1C). To assess the functional role of the iRhom1 re-entry loop in ADAM17 regulation, we generated a CRISPR-edited human epithelial osteosarcoma cell line (U-2 OS) lacking both iRhom1 and iRhom2, and measured ADAM17-dependent shedding of a chimeric alkaline phosphatase-transforming growth factor-α (AP-TGFα) reporter following reintroduction of iRhom1 cytoplasmic deletion constructs (Figure 2A). In all experiments, the cells were pre-incubated with the metalloproteinase inhibitor batimastat (BB94) to prevent substrate depletion by activating mutations. This precaution was included since activating iRhom mutations^47^ or Src mutations^48^ that increase basal ADAM17 activity can lead to premature substrate loss before stimulation, resulting in lower substrate pools at the start of the assay, which can obscure the detection of increased ADAM17 activity. After rapid BB94 washout, cells reconstituted with full-length iRhom1 or the Δ51 mutant showed low basal TGFα shedding, which was significantly enhanced upon stimulation with phorbol 12-myristate 13-acetate (PMA), a commonly used activator of protein kinase C (PKC) and stimulator of ADAM17 activity (Figure 2B).^12,22,49^ Deletion of 166 amino acids from the iRhom1 cytoplasmic domain (Δ166) led to elevated basal shedding that could be further enhanced by PMA, whereas the constitutive activities of Δ289 and Δ365 were even more strongly elevated and closely matched PMA-stimulated levels, with no further increase upon addition of PMA (Figure 2B). However, the larger Δ370 deletion, which removes a residue that is critical for stabilizing the re-entry loop (R369)^39^ completely abolished ADAM17 activity, both basal and PMA stimulated, underscoring the essential role of this structural feature (Figure 2B). Notably, all iRhom1 cytoplasmic deletion constructs were capable of supporting ADAM17 maturation and transport to the cell surface, a process dependent on iRhom binding to ADAM17 and facilitating its exit from the ER. However, constructs with larger deletions, such as Δ166, Δ289, Δ365, and Δ370, showed reduced levels of mature ADAM17 and lower cell surface expression compared to full-length and Δ51 iRhom1 (Figures 2C, 2D, and S4). The difference in stimulated shedding supported by full-length iRhom1 compared to the Δ166, Δ289, and Δ365-iRhom1 mutants is likely a result of reduced levels of mature ADAM17 on the cell surface. Importantly, while Δ365 promoted enhanced ADAM17-dependent TGFα shedding, Δ370 did not, despite both showing comparable ADAM17 maturation and surface expression (Figure 2). This suggests that the loss of activity observed in the Δ370-iRhom1 deletion mutant is likely not caused by the reduced maturation and surface levels but instead reflects disruption of a critical structural element, namely the re-entry loop.

**Figure 3.**
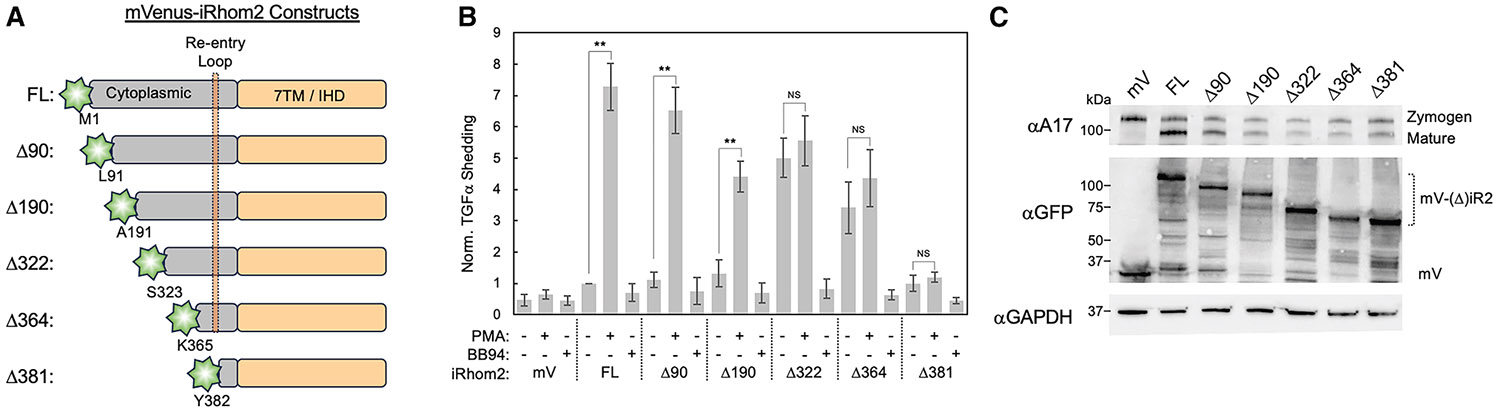
Function of the iRhom2 cytoplasmic region in ADAM17 activity (A) Schematic of the chimeric mVenus-iRhom2 cytoplasmic truncations, with mVenus depicted as a green star, iRhom2 shown with the cytoplasmic region in gray, and TM1-7 region in tan. (B) The re-entry loop is highlighted as an orange box. Deletion points and the first retained iRhom2 amino acid are noted. These constructs were assayed in ADAM17 shedding assays for TGFα after BB94 washout in iRhom1/2-null cells co-transfected with AP-TGFα. TGFα shedding was measured under basal shedding (untreated cells) or after the addition of either PMA or BB94. Data were normalized to the basal FL-iRhom2, and statistical significance was determined using an unpaired, two-tailed *t* test (where *p < 0.05; **p < 0.005; NS, not significant). Data are represented as the mean ± SD of *N* = 4 independent experiments. (C) Western blot analysis of ADAM17 (top) in iRhom1/2-null cells transfected with constructs from (A) (middle, αGFP). Zymogen and mature ADAM17 bands are indicated. αGAPDH (bottom) was used as a cell lysate loading control.

To further validate these observations, we tested previously characterized iRhom2 cytoplasmic deletion constructs in the same BB94 washout assay (Figure 3). Loss of the amino-terminal cytoplasmic region of iRhom2, up to residue S323 (Δ322) or K365 (Δ364), led to similarly increased basal TGFα shedding, mirroring the effects observed with the iRhom1 constructs (Figure 3B). Moreover, deletion of the re-entry loop (Δ381) in iRhom2 severely impaired TGFα shedding, while still supporting comparable levels of mature ADAM17 as Δ322 and Δ364 (Figures 3B and 3C). These observations reinforce the critical role of this structural element in both iRhom1 and iRhom2 in ADAM17 regulation.

### Structure-function studies of the iRhom1 TM region

**Figure 4.**
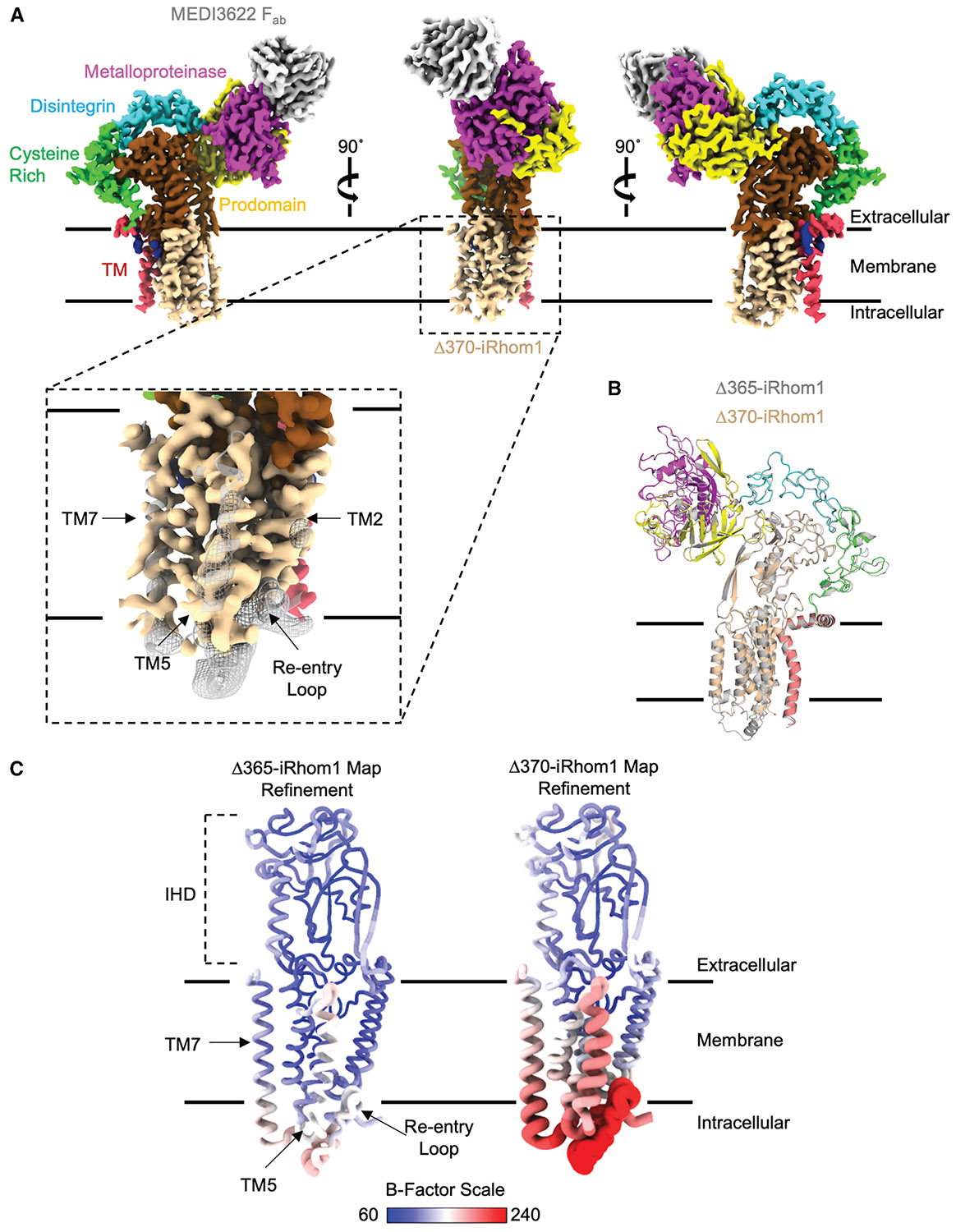
Structure of the zymogen ADAM17 bound by the re-entry-loop-deficient Δ370-iRhom1 complex (A) Cryo-EM map of the zymogen ADAM17-Δ370-iRhom1-MEDI3622 F_ab_ complex, colored by domain from Figure 1A embedded into the cell membrane, as indicated by parallel black lines, and putative lipid molecules are colored blue. (Inset box) A difference map (gray mesh) calculated between the Δ365-iRhom1 and Δ370-iRhom1 (colored) cryo-EM reconstructions highlights the absence of the re-entry loop feature and discontinuous signal associated with TM5 in the Δ370-iRhom1 map. The Δ365-iRhom1 model is shown as a gray cartoon. The re-entry loop, TM2, TM5, and TM7 are all noted with arrows. (B) Cartoon representation comparing the zymogen ADAM17 bound to either Δ365-iRhom1 (gray) or Δ370-iRhom1 (colored). (C) Ribbon representations of the zymogen ADAM17-Δ365-iRhom1 atomic model refined to the zymogen ADAM17-Δ365-iRhom1 cryo-EM map from Figure 1 (left) or to the zymogen ADAM17-Δ370-iRhom1 cryo-EM map in (A) (right). Color and radius of the ribbon representations scale to the atomic B-factor. The re-entry loop, TM5, TM7, and IHD are noted.

To better understand the atomic details of how the iRhom re-entry loop regulates ADAM17 function, we determined the cryo-EM structure of the Δ370-iRhom1 bound to zymogen ADAM17 in a GDN micelle to an overall resolution of 3.1 Å (Figures 4; and S5; Table S1). We reasoned that solving the Δ370-iRhom1 bound to ADAM17 structure could provide insights into regions of iRhom1 that regulate activation of ADAM17, since the Δ370-iRhom1 protein and Δ365-iRhom1 show indistinguishable trafficking and maturation, indicating proper folding, yet significantly differ in their ability to support ADAM17 activity. The resulting cryo-EM molecular reconstruction displayed well-resolved secondary structural features for all components. The previously determined zymogen ADAM17-Δ365-iRhom1 model and MEDI3622 F_ab_ were docked into the cryo-EM map, refined, and manually adjusted to optimize the fit (Table S1; Figure S6). Comparison of the Δ370 and Δ365 complexes revealed nearly identical overall architecture, both binding to zymogen ADAM17 and superimposing with an RMSD of 0.56 Å (Figure 4B). However, key differences were observed in the region of iRhom1 embedded in the membrane. Close inspection of the Δ370-iRhom1 cryo-EM map did not reveal features associated with the re-entry loop, despite the inclusion of this region in the expression construct (Figure 4A, inset). This suggests that deletion of R369 (discussed below), a conserved residue anchoring the iRhom re-entry loop to the base of the iRhom TM bundle, results in loss of this structural element likely due to increased conformational heterogeneity.^39^ Consequently, the iRhom1 TM5 appears as a discontinuous feature in the final cryo-EM map and elevated B-factors in the refined model, as well as a subtle ~2 Å shift toward TM2. Moreover, TM7 also exhibits higher B-factors, suggesting a broader destabilization of the iRhom1 TM bundle (Figure 4A, inset). To further assess these differences, the zymogen ADAM17-Δ365-iRhom1 atomic model was superimposed onto the Δ370-iRhom1 model and refined against the Δ370-iRhom1 cryo-EM map. Comparison of the resulting atomic B-factors revealed regions of increased atomic displacement in the Δ365-iRhom1 model when refined against the Δ370-iRhom1 cryo-EM reconstruction, which correlated with reduced local order in the cryo-EM map (Figure 4C). Importantly, the remaining transmembrane helices and the IHD displayed similar B-factor profiles between the two models, indicating that the overall fold is largely preserved. The observed structural differences were localized specifically to the re-entry loop, TM5, and TM7, supporting a destabilizing effect of the Δ370 deletion only on this region of the membrane domain of iRhom1. These findings suggest that the re-entry loop is critical for stabilizing the iRhom TM5 and TM7 in a conformation required for supporting ADAM17 enzymatic function.

**Figure 5.**
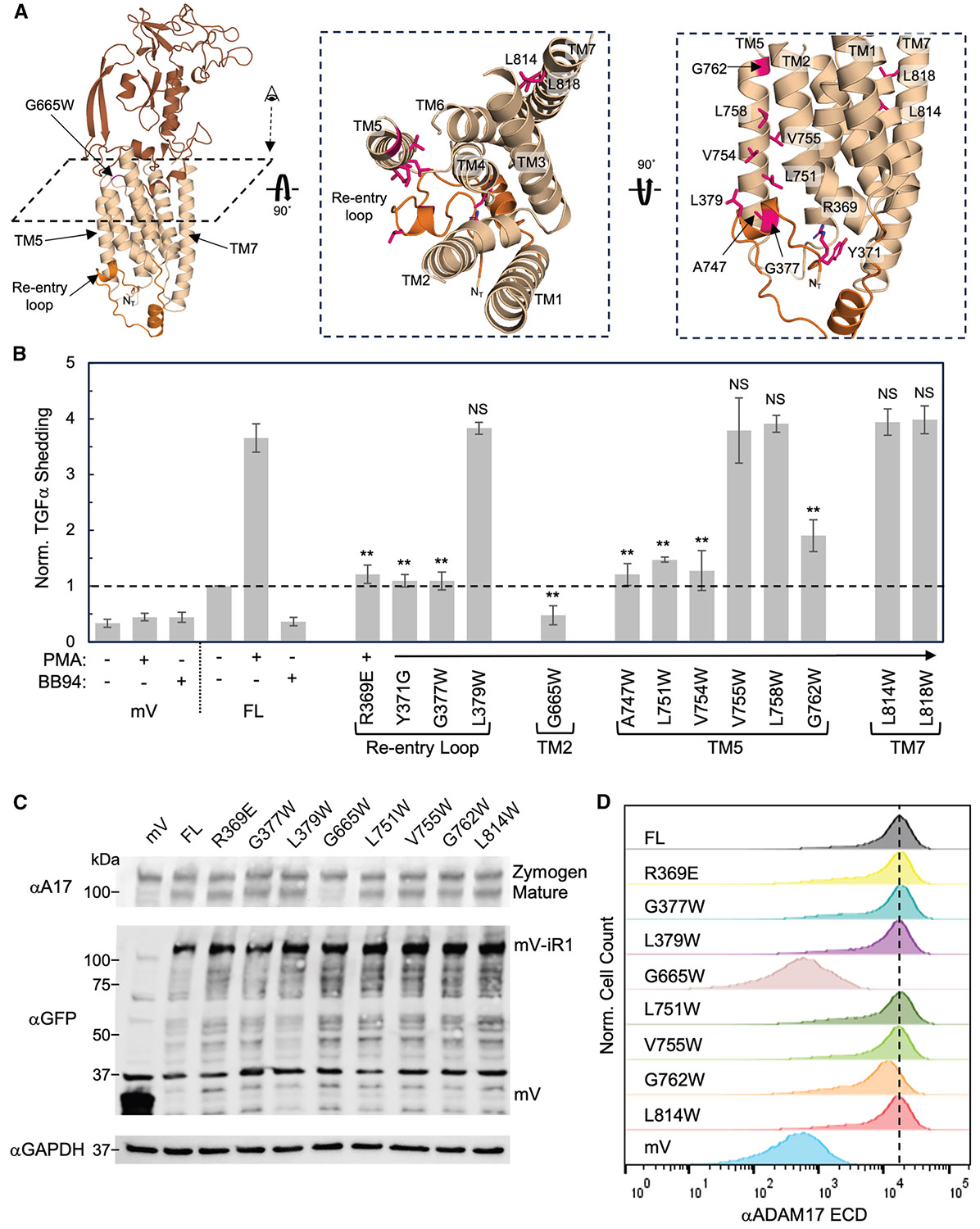
Functional characterization of the iRhom1 transmembrane region (A–C) Cartoon representation of iRhom1 with the re-entry loop colored orange, IHD brown, and TM regions in tan. Selected site-directed mutations within the TM region are labeled and represented as sticks (pink). These mutations were analyzed in (B) the ADAM17 TGFα shedding assay. TGFα shedding was measured after drug washout under basal shedding (untreated cells) or after addition of either PMA or BB94. Data were normalized to the basal iRhom1 (FL), and the effect of the individual mutations on PMA-stimulated shedding of TGFα was compared to PMA-stimulated iRhom1 (FL). Statistical significance was determined using an unpaired, two-tailed *t* test (where *p < 0.05; **p < 0.005; NS, not significant). Data are represented as the mean ± SD of *N* = 3 independent experiments. Selected iRhom1 mutations from (A) and (B) were analyzed by (C) western blotting analysis, assessing ADAM17 maturation (top, αA17). The iRhom expression was confirmed (middle, αGFP), and GAPDH (bottom, αGAPDH) served as a loading control. (D) Flow cytometry analysis of cell-surface ADAM17 using the ectodomain-specific antibody in the presence of the iRhom1 mutations from (C).

To investigate the mechanistic role of the iRhom1 TM5, TM7, and the re-entry loop in regulating ADAM17 activity, we introduced a series of point mutations into full-length iRhom1 and evaluated their effects using our cell-based shedding assay for AP-TGFα (Figure 5). We previously demonstrated that conserved arginine residues flanking the re-entry loop in iRhom2, particularly R367, were required to support elevated basal shedding of ADAM17 in cells expressing the Δ364-iRhom2 protein.^39^ To test the importance of the equivalent iRhom2 R367 residue in iRhom1, we introduced the charge-reversal mutation at R369E in the context of full-length iRhom1 (Figure 5A). We also identified a conserved tyrosine residue in the re-entry loop of iRhom1 and iRhom2 that fit optimally into the base of the rhomboid helical bundle, and tested its importance in stabilizing the re-entry loop by mutating Y371 to a glycine (Figure 5A). Additionally, we introduced a G377W mutation in the middle of the re-entry loop to sterically disrupt its spatial positioning relative to TM5. As a control, we included a L379W mutation, a residue buried in the membrane that is not predicted to perturb the rhomboid helical bundle. As anticipated, the L379W mutation had no effect on PMA-stimulated shedding of AP-TGFα compared to cells expressing iRhom1 (WT iRhom1) (Figure 5B). However, mutations targeting the re-entry loop (R369E, Y371G, and G377W) significantly reduced stimulated shedding, consistent with the re-entry loop having a critical role in regulating ADAM17 activation (Figure 5B).

The iRhom1 TM5 makes extensive hydrophobic contacts with the rhomboid core through interactions with the adjacent TM4 and TM6 helices (Figure 5A). To investigate the importance of TM5 in supporting ADAM17 activity, we introduced tryptophan substitutions along the rhomboid bundle-packing interface of TM5 (A747W, L751W, V754W, V755W, L758W, and G762W), increasing amino acid side chain size to disrupt local packing interactions. In addition, similar substitutions were introduced into TM7 (I804W, L814W, and L818W) (Figure 5A). When tested in our cell-based shedding assay, all TM5 mutations, except V755W and L758W, significantly impaired ADAM17-dependent shedding of TGFα compared to cells expressing WT iRhom1. By contrast, none of the TM7 mutations had a strong impact on stimulated TGFα shedding (Figure 5B). These observations further support the conclusion that the precise architecture of the iRhom TM bundle, particularly the positioning of TM5, is essential for promoting ADAM17 catalytic activity.

Importantly, all iRhom1 variants, including those with impaired ADAM17 activity, still supported protease maturation and trafficking to the cell surface as confirmed by immunoblotting and flow cytometry analysis, respectively (Figures 5C, 5D, and S7). These results indicate that the observed loss of activity is not due to an effect on ADAM17 maturation or surface localization. Rather, the structural perturbations localized to the re-entry loop and TM5 appear to directly compromise the activation mechanism of ADAM17. To further explore this model, we examined a recessive disease-associated iRhom1 mutation, G665W, located in TM2, which was identified in a patient with cardiomyopathy.^50^ This variant not only failed to support PMA-stimulated shedding of TGFα in our cell assays, but it also exhibited pronounced defects in ADAM17 maturation and surface trafficking (Figures 5B-5D and S7). These findings suggest that the G665W mutation, unlike those tested in the re-entry loop and TM5, likely destabilizes the rhomboid fold, causing iRhom1 to misfold or to be unable to form a proper complex with ADAM17 that allows exit from the ER and transport to the cell surface.

**Figure 6.**
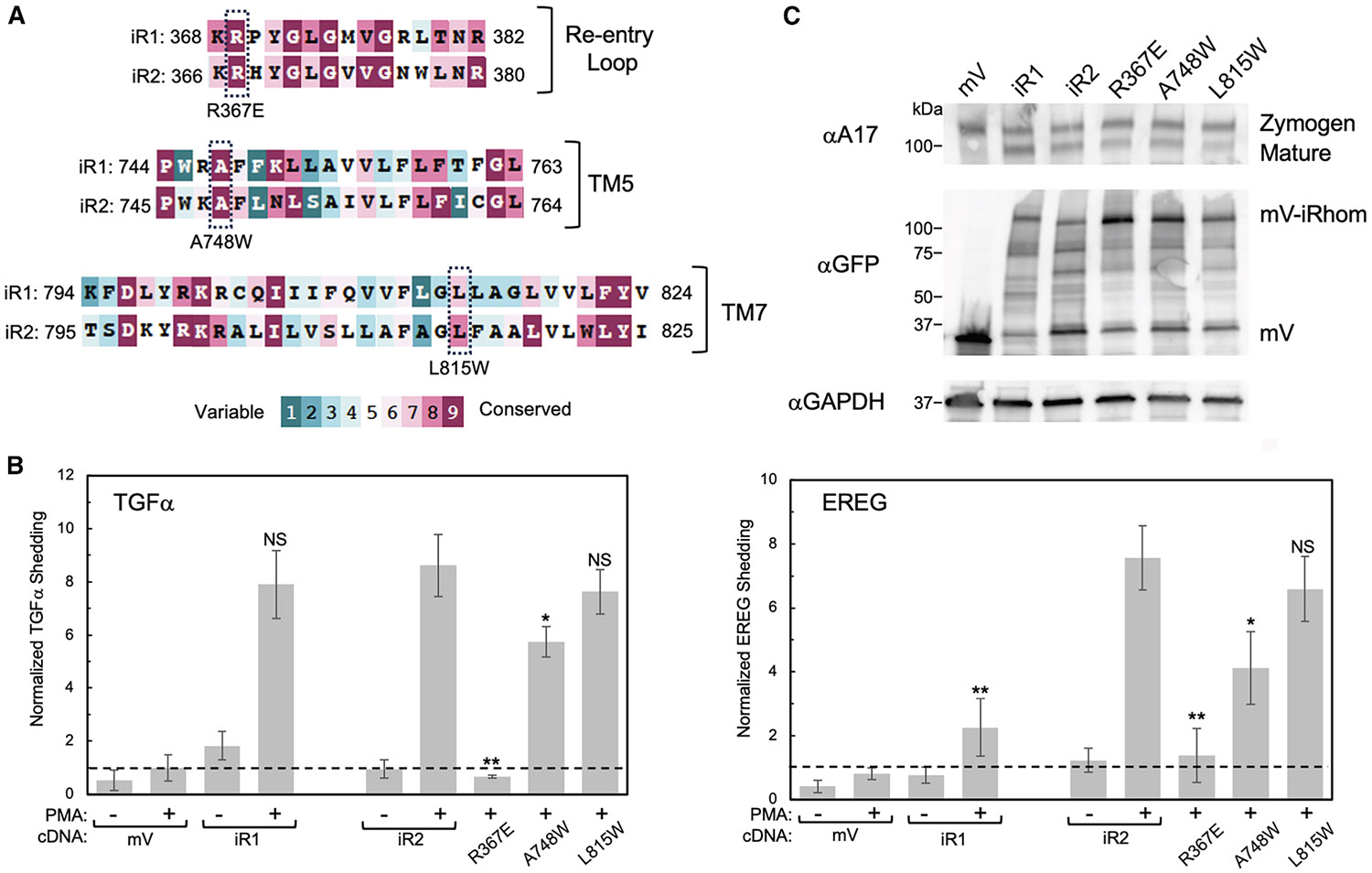
Re-entry loop and TM5 structural features broadly regulate ADAM17 substrate processing (A and B) Sequence alignment of the iRhom1 and iRhom2 re-entry loop, TM5 and TM7, colored by conservation scores calculated using the Consurf server.^52^ Dashed boxes indicate the positions of single-point mutations in iRhom2 analyzed in the ADAM17-dependent shedding assay in (B), TGFα (left), and EREG (right). Substrate shedding was measured after drug washout under basal conditions (untreated cells) or after addition of PMA (+). Data were normalized to the basal iRhom2 (iR2), and the effects of the individual mutations on PMA-stimulated shedding were compared to PMA-stimulated iRhom2 (iR2 +PMA). Statistical significance was determined using an unpaired, two-tailed *t* test (where *p < 0.05; **p < 0.005; NS, not significant). Data are represented as the mean ± SD of *N* = 3 independent experiments. (C) Western blotting analysis of cells expressing the indicated iRhom mutations, assessing ADAM17 maturation (top, αA17). iRhom expression was confirmed using αGFP (middle), and GAPDH (bottom, αGAPDH) served as a loading control.

ADAM17 substrate selectivity depends in part on the associated iRhom paralog, with the iRhom2 TM7 previously implicated in regulating selective shedding of substrates such as EREG.^51^ Notably, the re-entry loop and TM5 do not make direct contact with TM7, raising the possibility that these regulatory features function independently of the substrate-selective TM. To address this question, we generated selected mutations in iRhom2 that correspond to those analyzed in iRhom1, targeting the re-entry loop (R367E), TM5 (A748W), and TM7 (L815W), and measured ADAM17-dependent shedding of TGFα and the iRhom2 selective substrate EREG using our cell-based assay (Figure 6). Mutation of the iRhom2 re-entry loop (R367E) strongly attenuated stimulated shedding of both TGFα and EREG, whereas the disruption of TM5 (A748W) produced a more modest reduction in shedding. In contrast, the TM7 mutation (L815W), which is not predicted to interfere with substrate-selective interactions, fully supported ADAM17-mediated shedding of both substrates. Importantly, the reduced shedding observed for the iRhom2 re-entry loop and TM5 mutations occurred in the absence of defects in ADAM17 maturation (Figure 6C). Collectively, the re-entry loop and TM5 define a conserved activation axis that regulates the activation of ADAM17-dependent shedding independently of its substrate selectivity, which is instead mediated by separate iRhom and ADAM17 structural features, such as the TM1 and TM7 domains.^40,41,51^

### MD modeling of the mature ADAM17-iRhom1 complex

**Figure 7.**
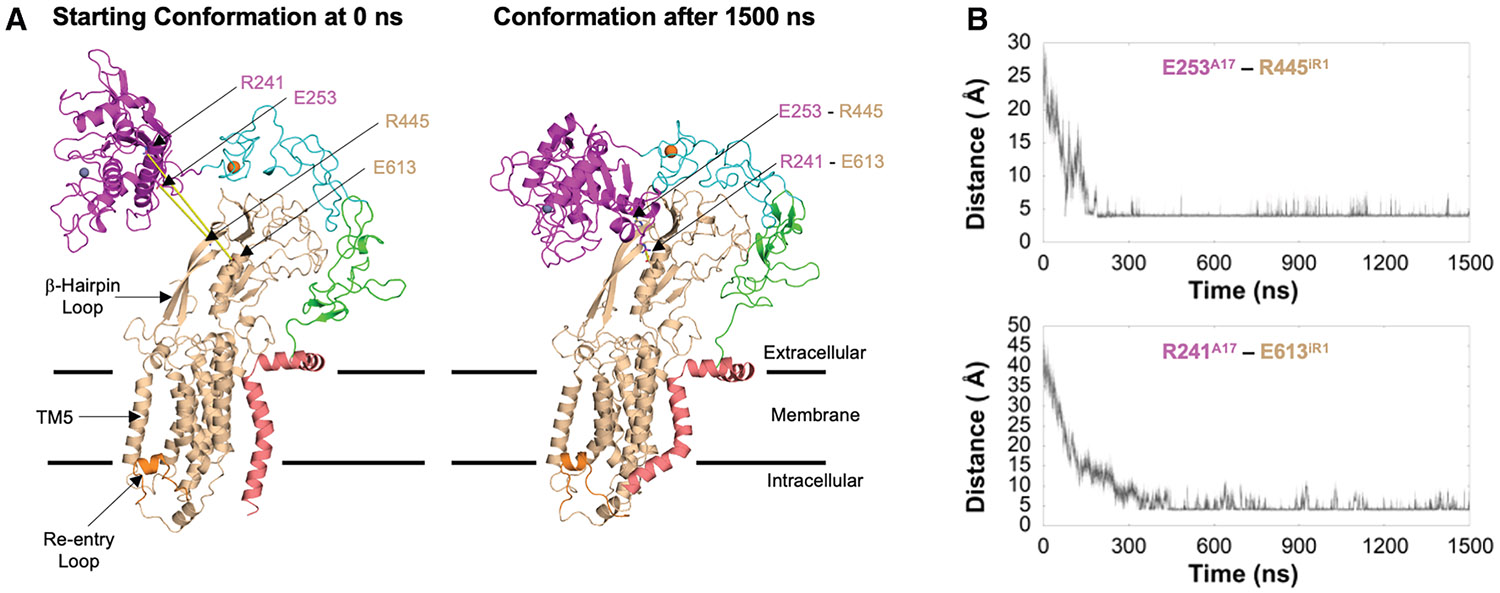
All-atom molecular dynamics simulation of the maturation of the ADAM17-iRhom1 complex (A) Cartoon representation of the mature ADAM17-iRhom1 (prodomain removed), with the remaining domains colored as described in Figure 1. The starting conformation at 0 ns (left) is compared to the resulting conformation after 1,500 ns of MD simulation (right) in the same orientation. (B) Time evolution distances of amino acid pairs in the M domain and IHD (E253-R445 and R241-E613, connected by yellow dashed lines in A), plotted as distance (Å) over the course of the 1,500 ns MD simulation.

The observation that mutations in the re-entry loop and in TM5 of iRhom proteins affect the activation of mature ADAM17 raises important questions about the position of the M domain after removal of the inhibitory pro-domain. Previous functional studies, confirmed here, have demonstrated that ADAM17 exhibits distinct substrate selectivity depending on its association with endogenous iRhom1 or iRhom2,^35,40,41,51^ suggesting that the M domain of ADAM17 interacts closely with the IHD of iRhom1 or iRhom2. To explore potential conformational changes of the M domain after removal of the pro-domain, we performed unbiased all-atom MD simulations to gain mechanistic insights into the maturation process of ADAM17. For the starting model, we placed the zymogen ADAM17-Δ365-iRhom1 into a hydrated lipid bilayer and removed the pro-domain at the pro-protein convertase cleavage site (Arg214/Arg215).^18^ Upon prodomain removal, the M domain transiently sampled different positions relative to the IHD for ~200 nanoseconds before establishing a stable interaction to the tip of the β-hairpin loop of the IHD, as measured by the distances between two residue pairings (M domain residues E253 to IHD R445, and M Domain R241 to IHD E613) (Figure 7; Video S1). This interaction persisted for the remainder of the 1.5 μs simulation and was maintained through a combination of electrostatic and hydrophobic interactions, overlapping the same region occupied by the pro-domain in the zymogen structure (Figure 7; Video S1). To complement these simulations, we used AlphaFold3 (AF3) to model the mature ADAM17 bound to iRhom1 (Figure S8). The resulting AF3 models also positioned the M domain in contact with the β-hairpin of the IHD, and structural alignment (using the structure of iRhom1) with the final MD structure revealed similar binding interfaces between the IHD and the M domain, although in the AF3 model the M domain is rotated by ~30° along an axis parallel to the cell membrane and tilted slightly toward it. The β-hairpin is positioned above the TM5 in the rhomboid bundle, placing the M domain in register with these regulatory transmembrane elements. The functional importance of the TM5 and re-entry loop positioning is further supported by additional MD simulations comparing mature ADAM17-iRhom1 AF3-derived models with and without the re-entry loop, which revealed significant movement of TM5 within the rhomboid bundle and loss of a cytoplasmic helical segment preceding TM1 upon removal of the re-entry loop, recapitulating the structural features observed in the Δ370-iRhom1 cryo-EM structure (Figure S9; Videos S2 and S3). Taken together, these independent computational approaches support a hypothetical model in which, following prodomain removal, the M domain makes contact with the β-hairpin of the IHD and is positioned in line with re-entry loop and TM5, forming a structural axis that supports ADAM17 activation.

## DISCUSSION

Here, we report the cryo-EM structure of the ADAM17 zymogen bound to iRhom1, revealing atomic details that govern the complex formation and regulate protease function. We further demonstrate that the cytoplasmic domains of iRhom1 and iRhom2 have analogous roles in controlling the stimulated shedding of ADAM17 substrates in cell-based assays. Moreover, through additional structural analysis and functional studies, we identify a critical iRhom1 TM helix that links the intracellular signaling inputs to the activation of ADAM17 at the cell surface. Finally, based on all-atom MD simulations, we propose a hypothetical model for the maturation of ADAM17 in complex with iRhom1.

The results presented here highlight the role of a key functional element, the cytoplasmic structural feature termed the “re-entry loop,” initially discovered in iRhom2^38,39^ in the post-translational regulation of ADAM17 by iRhom1 and iRhom2. The re-entry loop is a highly conserved structural element, anchored at the base of the rhomboid fold, wedged between TM5 and TM2. Our previous studies have highlighted the importance of this domain in regulating ADAM17 activity in iRhom2.^39^ Here, we demonstrate that the equivalent structural element in iRhom1 also critically controls stimulated ADAM17-dependent substrate shedding. Mutagenesis of the R369 residue, anchoring the re-entry loop to the rhomboid fold, or sterically repositioning this loop using a G377W mutation, resulted in a dysfunctional iRhom1 protein incapable of supporting the stimulation of TGFα shedding by ADAM17, though both mutants still retained the ability to promote ADAM17 maturation and facilitate its trafficking to the cell surface. The critical anchoring function of R369 is exemplified by our second cryo-EM structure, in which deletion of this residue led to the re-entry loop being unresolved and TM5 appearing discontinuous within the reconstructed ADAM17-iRhom1 complex map. This structural perturbation implicates TM5 as a molecular bridge, connecting intracellular regulatory signals to ADAM17 enzymatic activity on the extracellular side of the membrane. Indeed, disrupting the precise positioning of TM5 through targeted single-point mutations dramatically reduced ADAM17 activity, without altering protease maturation or its trafficking to the cell surface. Collectively, these results support a model in which the re-entry loop and TM5 act as a molecular relay, transmitting information across the cell membrane to regulate ADAM17 catalytic function in response to intracellular signaling events.

In contrast, not all mutations we examined exhibited this selective dysfunction in iRhom1. Notably, we evaluated an iRhom1 missense mutation, G665W, identified in a young patient diagnosed with cardiomyopathy.^50^ This mutation is located at the junction between the IHD and TM2 and likely induces steric repositioning or destabilization of the rhomboid fold. When screened in our cell-based assays, we found that this mutation, like several others, was unresponsive to stimulation with PMA. However, unlike the mutations in the re-entry loop and TM5 that retained maturation capability, the G665W variant showed no evidence of mature ADAM17 and apparently failed to traffic to the cell surface. This suggests that G665W causes a broader disruption of iRhom1 assembly with ADAM17 and likely results in misfolding. This may explain why the cardiomyopathy associated with iRhom1-G665W resembles that seen in two patients with a frame-shift mutant in iRhom1 (F405Sfsrter16) that truncates the coding sequence just N terminal to TMD1, likely resulting in a complete loss of iRhom1-dependent ADAM17 activity.^50^

The cytoplasmic regions of iRhom1 and iRhom2 are essential for interpreting intracellular signals that control the stimulated shedding of ADAM17 substrates from the cell surface. In particular, patients carrying P189L or I186T mutations in the cytoplasmic domain of iRhom2 are predisposed to develop palmoplantar hyperkeratosis and tylosis with esophageal cancer.^53-56^ This region is conserved between iRhom1 and iRhom2, and a knockin mouse model has demonstrated that one of these mutations (the mouse equivalent to P189L) confers gain-of-function activity for ADAM17, particularly dependent on amphiregulin (AREG) signaling.^57,58^ In addition, a large cytoplasmic deletion in iRhom2, referred to as the *cub* mutation,^59,60^ also leads to an ADAM17-dependent gain-of-function phenotype in mice.^58^ Here, using our cell-based shedding assay with BB94 washout, we show that the deletion of the cytoplasmic region encompassing the *cub* mutation in both iRhom1 (Δ289) and iRhom2 (Δ322) greatly increases basal shedding of TGFα by the expressed proteins but eliminates any further enhancement upon PMA stimulation. These data suggest that the regions of the iRhom cytoplasmic domain that are N terminal in iRhom1 (Δ289) and iRhom2 (Δ322) contain sequences that can mediate negative regulation of ADAM17 activity. Moreover, additional larger cytoplasmic deletions further reinforce the observation that the re-entry loop is a conserved structural feature in both iRhom1 and iRhom2 and is necessary for ADAM17 activation.

Although the deletion of the iRhom cytoplasmic N-terminal region increases constitutive ADAM17 shedding, it also results in a general reduction in ADAM17 maturation and trafficking to the cell surface. This likely reflects the requirement for additional regulatory proteins that facilitate forward trafficking of the ADAM17-iRhom complex. Notably, these activating mutations also remove a region of the iRhom cytoplasmic domain that contains the FERM domain-interacting motif, which is required for binding FRMD8, a protein known to promote ADAM17-iRhom complex formation and stability.^43,44,46^ Accordingly, FRMD8 knockout mice exhibit little mature ADAM17,^46^ supporting the idea that FRMD8 is crucial for proper assembly and ER exit of the ADAM17-iRhom2 complex. We observe that deletion of the first 166 amino acids begins to show defects in stimulated shedding and forward trafficking of the ADAM17-iRhom1 complex. It is possible that other regulators, such as 14-3-3 adaptor proteins, might still bind to and regulate intracellular signaling pathways.

Finally, from our computational description of the mature ADAM17 bound to iRhom1, the relevance of the β-hairpin of the IHD of iRhom1 in modulating the positioning of the M domain was evident. Even though the β-hairpin of the IHD of iRhom1 links TM1 with the rest of the IHD, it is positioned on top of TM2 and TM5. It is tantalizing to speculate that this communication pathway may serve to connect structural changes from the intracellular side to the TM domain (re-entry loop and TM5). This could then conceivably have an allosteric effect on the ECD of iRhom1 (β-hairpin in the IHD) and the M domain of ADAM17 that could affect its function and substrate selectivity. Future studies will be necessary to further explore this model.

### Limitations of the study

Despite the advances presented in this study, certain limitations remain. Humans express two iRhom paralogs, iRhom1 and iRhom2, which share considerable sequence identity but have also been shown to have distinct effects on the substrate repertoire of ADAM17 under stimulated conditions.^35,51^ This raised the possibility that iRhom1 and iRhom2 might engage ADAM17 differently, resulting in distinct structural features. However, the comparison of the cryo-EM structures of zymogen ADAM17 bound to either iRhom1 (this study) or iRhom2^39^ reveals an almost identical contact interface. Thus, the mechanism underlying the different substrate selectivity of iRhom1-ADAM17 versus iRhom2-ADAM17 remains to be established. For this, the structure of a complete mature ADAM17-iRhom complex will need to be determined, and possible differences in allosteric conformational changes or interactions with yet to be identified additional co-factors could be responsible for the different substrate selectivity. Moreover, the proposed allosteric changes in the ECD of the ADAM17-iRhom2 complex that stimulate its catalytic activity and that are regulated by the re-entry loop will likely only become evident in the complex with mature ADAM17. MD simulations and predictive AlphaFold3 models place the mature ADAM17 catalytic (M) domain in close proximity to the iRhom re-entry loop and TM5, which we identify here as forming a molecular relay between the intracellular and extracellular environments. Importantly, these computationally derived models are hypothetical and subject to experimental validation, especially since they differ from previously proposed models based on cryo-EM data, which were interpreted to indicate that the M domain is highly mobile and sampling multiple conformations, at least in complex with iRhom2.^38^ Part of the difficulty in resolving the mature ADAM17-iRhom complex by cryo-EM may stem from the weak interactions predicted between the M domain and IDH that can be sensitive to disruption by classical cryo-EM sample preparation methods, which can destabilize low-affinity protein-protein interactions.^61^ Moreover, TM7 in iRhom2 has been implicated in regulating the stimulated shedding of the iRhom2-selective substrate EREG but does not affect TGFα shedding,^51^ yet how it functions in concert with other structural elements to determine the substrate selectivity of the ADAM17-iRhom complexes remains poorly understood. Future efforts to resolve substrate-bound ADAM17-iRhom complexes will be critical for revealing the mechanistic basis of substrate recognition and selective shedding. Finally, our cryo-EM structures lack the cytoplasmic regions of iRhom1, which we have shown to be essential for regulating ADAM17 activity and the precise inflection points within the iRhom cytoplasmic domain that govern stimulated shedding, potentially as a negative regulator, of ADAM17 remain undefined. The underlying molecular mechanisms are also unknown and represent an area of active investigation.

## STAR★METHODS

### EXPERIMENTAL MODEL AND STUDY PARTICIPANT DETAILS

#### Cell lines

Each cell line used in this study (U-2 OS, Expi293F, and Sf9) and their respective growth media have been previously described.^39^ All cell lines were maintained using the suppliers’ handling recommendations. The generation of the double KO *RHBDF1*^−/−^ and *RHBDF2*^−/−^ in the U-2 OS cell line was performed by the Cincinnati Children’s Hospital Medical Center Transgenic Animal and Genome Editing Core Facility, using CRISPR-Cas9 gene editing methods. Specifically, CRISPR targeting constructs to the exon 3 of the *RHBDF1,* and exon 5 of the *RHBDF2* gene loci were introduced to make genomic deletions, resulting in knockout of the genes of interest. Successful genetic deletions of both iRhom genes in the single cell clones were analyzed by sequencing. All cell lines were confirmed to be mycoplasma free and authentication was verified from the suppliers. Sex of cell lines was not considered a variable in this study.

### METHOD DETAILS

#### Cloning

ADAM17 and MEDI3622 antibody plasmids were generated as described previously.^39^ All iRhom1 expression constructs were PCR-amplified from a cDNA and sequentially subcloned with an FLAG tag sequence linker into pEYFP-mVenus-C1 vector (Addgene: Plasmid #27794), followed by cloning into pEG-BacMam as mVenus-iRhom1 chimeric proteins. Viruses were then generated using the Bac-to-Bac Expression system (Thermo Fisher Scientific). Single point mutations within iRhom1 and iRhom2 that were used in the AP-TGFα cell shedding assay were generated using site directed mutagenesis in the pEYFP-mVenus-iRhom1 plasmid.

#### Purification

Purification of the iRhom1-ADAM17 complexes was carried out in Expi293 cells that were co-infected with baculoviruses designed to express either ADAM17(E/A) or chimeric mVenus-iRhom1 proteins using the Bac-Mam expression system and supplemented with 10 μL/mL of 45% glucose and 300 mM Valproic Acid. After 72 h, cells were homogenized with a 1% LMNG buffer containing 30% glycerol, 20 mM HEPES pH 8.0, 300 mM NaCl, 1 mM CaCl, and 0.1% cholesteryl hemisuccinate (CHS). This buffer was supplemented with 2 mg/mL iodoacetamide, and one tablet of Pierce Protease Inhibitor (ThermoFisher). Clarified lysate was then purified using a CNBr-GFP Nanobody affinity column, where the detergent was buffer exchanged for a 0.01% glyco-diosgenin (GDN) buffer. After elution from the affinity column, the complex was mixed with excess amounts of the MEDI3622 F_ab_ and isolated using size exclusion chromatography on a Superose 6 Increase 10/300 GL column. Fractions were assayed via SDS-PAGE for homogeneity, pooled and concentrated using Millipore Amicon Ultra 100k centrifugal filters for immediate cryo-EM grid preparation.

#### Cryo-EM sample preparation and data acquisition

The ADAM17-Δ365-iRhom1 and ADAM17-Δ370-iRhom1 in complex with the MEDI3622 F_ab_ were concentrated to 8.0 mg/mL and 10.0 mg/mL, respectably. These samples were individually applied to AltrAuFoil R 1.2/1.3 grids that were glow discharged using a PELCO easiGlow Glow Discharge Cleaning System; 0.39 mBar, 20 mA, glow time/hold 30/10 s. Grids were blotted with a blot force setting between 1 and 10, for times ranging 1–10 s and plunge frozen in liquid ethane using a Vitrobot Mark IV System.

All ADAM17-iRhom1 grids were screened on a 200 kV Glacios TEM at the University of Cincinnati School of Medicine Center for Advanced Structural Biology and higher resolution datasets were collected at the Vanderbilt School of Medicine Center for Structural Biology on a FEI Titan Krios operated at 300 kV with a K3 BioQuantum direct electron detector camera in counting mode. Datasets were collected at a nominal magnification of 130,000× with a pixel size of 0.647 Å. Individual datasets for ADAM17-Δ365-iRhom1 and ADAM17-Δ370-iRhom1 were collected using a total dose of 63.5 e^−^/Å^2^ and 56.2 e^−^/Å^2^, respectively, that were used for single particle analysis structure determination.

#### Cryo-EM single particle analysis and model building

All cryo-EM data were processed using CryoSPARC on the GPU cluster maintained by the University of Cincinnati Advanced Research Computing Center and all cryo-EM software, excluding CryoSPARC, was maintained by the SBGrid research computing consortium. Movies were motion-corrected using patch motion correction, and contrast transfer function (CTF) parameters were estimated with patch CTF estimation. Corrected movies were curated based on total full-frame motion distance (below 40 pixels), relative ice thickness (below 1.1), and CTF fit resolution (below 4.5 Å). This curation resulted in 38,428 and 5,449 movies used for single particle analysis of the ADAM17-Δ365-iRhom1 and ADAM17-Δ370-iRhom1 structures, respectively.

For the ADAM17-Δ365-iRhom1 structure, initial particles were picked using a Laplacian of Gaussian auto picking approach on a subset of 1000 curated movies to generate template 2D classes for picking on the same subset of corrected movie files. After iterative rounds of pruning particles, using 2D classification, an *ab-initio* model was created that provided a modest resolution structure of the ADAM17-iRhom1 complex bound by the MEDI36221 F_ab_. Particles from this model served as a reference for training the Topaz particle-picking model, which subsequently identified particles from the 38,428 curated movie set. After curating this particle stack through 2D classification, ~5.6 × 10^6^ particles were subjected to three rounds of heterogeneous refinement using four molecular models and a single 3D classification improved the model resolution to 5.37 Å from a particle stack containing 96,377 images. To further increase the model’s resolution to 3.52 Å, sequential *ab-*initio models were generated and improved using non-uniform refinement and local refinement jobs. Application of cryoSPARC’s 3dFlex procedures revealed significant movement of the MEDI3622 F_ab_ constant domain and local refinement procedures,^62^ masking the MEDI3622 F_ab_ constant domain, yielded the final cryo-EM map at 3.37 Å. The overall workflow is depicted in Figure S1. The zymogen ADAM17, MEDI3622 F_ab_ models (RCSB PDB ID:9O58) and Δ365-iRhom1 AlphaFold3 predictive model were fit into the B-factor sharpened cryo-EM map using ChimeraX. The final model was built in Coot and refined in Phenix Real-Space Refine. Model to map FSC was produced using Phenix Comprehensive Validation (Table S1). The final local refined map was post-processed with DeepEMhancer using the highRes algorithm for figure creation.

For the ADAM17-Δ370-iRhom1 structure, initial particle stacks were generated using a Laplacian of Gaussian auto picking approach followed by two rounds of 2D classification to identify set of 2D references for use in a Template based picking approach. After pruning the particle stack with two rounds of 2D classification the resulting ~1.x10^6^ particle images were sorted using a heterogeneous refinement job with four models, one a low pass filtered map from the ADAM17-Δ365-iRhom1 workflow. The sorted particles into the ADAM17-iRhom1-MEDI3622 F_ab_ complex were improved using 3D classification to a resolution of 4.91 Å. The 51,656 particles were analyzed using 2D classification and used as a reference for training the Topaz particle-picking model from the 5,449 curated movie files. The particle stack was pruned using the 2D classification, heterogeneous refinement (4 models) and 3D classification to yield a 3.42 Å model with 68,712 particles. The resolution was improved to 3.17 Å using two iterative *ab-initio* model generating jobs, removing any model bias, followed by non-uniform and local refinement jobs, making the F_ab_ constant domain as significant movement was observed for this domain in the 3DFlex output. The overall workflow can be found in Figure S5. The ADAM17-Δ365-iRhom1 model was fit into the B-sharpened cryo-EM map using ChimeraX, built in Coot and refined in Phenix Real-Space Refine. Model to map FSC was produced using Phenix Comprehensive Validation (Table S1). The final local refined map was post-processed with DeepEMhancer using the highRes algorithm for figure creation.

#### Cell-based ADAM17-dependent TGFα shedding assay

The enzymatic activity of ADAM17 was assessed using the metalloproteinase inhibitor Batimastat (BB94) in a drug washout assay, adapted from previously described protocols.^12,23,39,47,63^ In this approach, iRhom1/2-null U-2 OS cells were transfected in 6-well plates (1 × 10^6^ cells/mL) with 750 ng of an alkaline phosphatase (AP)-tagged TGFα and 375 ng of the mVenus-iRhom1 or mVenus-iRhom2 constructs in media containing 5 μM BB94, thereby preventing ADAM17 activity and possible substrate depletion by constitutively active mutants until the inhibitor-containing media was removed. After 48 h, cells were washed with DMEM to remove residual serum, and media was replaced with one of the following: DMEM alone, DMEM with 5 μM BB94, or DMEM with 25 ng/μL phorbol 12-myristate 13-acetate (PMA). Following a 1 h incubation, conditioned media and cell lysates were collected separately and analyzed for alkaline phosphatase conversion of 4-nitrophenyl phosphate (PNPP, New England Biolabs) at 405 nm over 90 min using a BioTek SynergyH1 plate reader as previously described.^12,23,63^ Individual reaction slopes were calculated, and ADAM17 shedding activity was expressed as the supernatant rate divided by the sum of the supernatant rate plus lysate rate. All results were normalized to DMEM-treated full-length wild-type iRhom controls under basal shedding conditions.

#### Western blot

To assess expression levels (mVenus-iRhom, ADAM17, GAPDH) and maturation (ADAM17) of proteins used in this study, we performed western blot analysis for each respective construct. Briefly, 1 μg of mVenus-iRhom constructs were transfected and analyzed by Western Blot for the presence of mVenus-iRhom protein and ADAM17 maturation, where GAPDH was used as a loading control. Primary antibodies used to generate blots were as follows: αADAM17 (Abcam, Ab39162), αGFP (BioRad, AHP975), αGAPDH (Cell Signaling, D16H11).

#### Flow cytometry

*RHBDF1*^−/−^ */RHBDF2*^−/−^ U-2 OS cells were seeded in 6-well plates at 70% confluency and transfected with 1 μg of iRhom expression constructs using PEI (1 mg/mL) at a 3:1 PEI:DNA ratio. After 48hrs, cells were trypsinized and pelleted by centrifugation at 700 rpm for 5 min and suspended in PBS. Cells were stained for ADAM17 extracellular domain (ECD) by incubation with a conjugated Alexa Fluor 647-labeled αADAM17-ECD antibody (R&D Systems, FAB9301R) at a 1:100 dilution for 1 h at room temperature in PBS supplemented with 0.5% w/v Bovine Serum Albumin. Cells were then washed with 10x volume of PBS and suspended in 300 μL of PBS for flow cytometry analysis. Flow cytometry was performed using a Cytek Aurora spectral flow cytometer. Single-stain controls included non-transfected *RHBDF1*^−/−^ */RHBDF2*^−/−^ cells stained with the αADAM17-ECD. Cells transfected with the either mVenus (control) or the mVenus-iRhom constructs were gated for the intrinsic fluorescence of mVenus (excitation at 488 nm, emission collected at 530/30 nm), and Alexa Fluor 647-conjugated αADAM17-ECD was used to assess the levels of ADAM17 on the cell surface (excitation at 640 nm, emission collected at 660/20 nm). Spectral unmixing was conducted using single-stain controls to ensure accurate signal separation. Data acquisition was performed with Cytek SpectroFlo software and analyzed using FlowJo.

#### Unbiased all-atom molecular dynamics simulations

To investigate the mature catalytic domain of ADAM17, the cryo-electron microscopy (cryo-EM) structure of iRhom1 bound to the zymogen ADAM17 (including the A406E catalytic site inactivating mutation) was utilized where the pro-domain was removed at the pro-protein convertase cleavage site (R214 ↓ R215). The resulting iRhom1/ADAM17 complex was hydrated with TIP3P water molecules and embedded in a lipid bilayer constituted by 1-palmitoyl-2-oleoyl-*sn*-glycero-3-phosphocholine (POPC) molecules; both ions, Zn and Ca, observed in the cryo-EM structure, were also included. The final system, constituted by about 170,000 atoms, was investigated by all-atom molecular dynamics (MD) simulations performed using constant values for the temperature (37°C), pressure (1 atm) and salt concentration (0.15 M NaCl).^51,64^ The system was subjected to preliminary treatment including minimization and equilibrium phases to remove possible atom clashes and produce adequate lipid packing around the protein complex.^65,66^ Next, three restriction phases were performed, applying a harmonic potential in the atoms of 1.0, 0.5, and 0.1 kcal/mol A2, respectively. Finally, the system was investigated for 1.5 μs unbiased simulations using a 2.0 fs time step.

### QUANTIFICATION AND STATISTICAL ANALYSIS

Flow cytometry-based surface staining (mean fluorescence intensity) and cell-based AP shedding assays (normalized to basal shedding conditions) were analyzed in Microsoft Excel using an unpaired, two-tailed *t* test. Statistical significance is indicated by asterisks with corresponding *p* values, as described in the figure legends. All data are presented as mean values ±standard deviation (SD) from 3 to 4 independent experiments, as specified in the figure legends.

## Supplementary Material

1

2

3

4

Supplemental information can be found online at https://doi.org/10.1016/j.celrep.2026.117309.

## Figures and Tables

**Table:** KEY RESOURCES TABLE

REAGENT or RESOURCE	SOURCE	IDENTIFIER
Antibodies		
MEDI3622 recombinant monoclonal antibody	This study	N/A
Anti-hTACE Alexa Fluor 647 Conjugated Mouse igG	R&D Systems	Cat#FAB9301R; RRID: AB_2223551
Rb pAb to ADAM17	Abcam	Cat#ab39162; RRID: AB_722565
Goat Anti-Green Fluorescent Protein	BioRad	Cat#AHP975; RRID: AB_566990
GAPDH (D16H11) XP Rabbit mAb	Cell Signaling	Cat#8884; RRID: AB_11129865
Goat anti-Human IgG Fc Secondary Antibody, Alexa Fluor 647	Thermo Fisher Scientific	Cat#A55749; RRID: AB_2925774
Bacterial and virus strains		
E. coli Stellar Competent Cells	Takara Bio	Cat#636763
Bac-to-Bac baculovirus system	Thermo Fisher Scientific	Cat#A11100
BacMam-ADAM17	This Study	N/A
BacMam iRhom1	This Study	N/A
EMBacY bacmid strain	Berger et al., 2004	N/A
Chemicals, peptides, and recombinant proteins		
Phorbol 12-myristate 13-acetate (PMA)	Sigma Aldrich	Cat# P8139
LMNG	Anatrace	Cat# NG310
GDN	Anatrace	Cat# GDN101
Cholesteryl Hemisuccinate, Tris Salt	Anatrace	Cat# CH210
Iodoacetamide, 98%	Thermo Fisher Scientific	Cat#122270250
Pierce Protease Inhibitor Tablets, EDTA-Free	Thermo Fisher Scientific	Cat# A32965
FLAG peptide	Sigma-Aldrich	Cat# F3290
Recombinant ADAM17	This Study	N/A
Recombinant iRhom1 and iRhom2	This Study	N/A
*p*-Nitrophenyl Phosphate (pNPP) Substrate	New England Biolabs	Cat# P0757
Batimastat (BB94) metalloproteinase inhibitor	Tocris Bioscience	Cat# 2961
Critical commercial assays		
Gibson Assembly Master Mix	New England Biolabs	Cat# E2611
Lipofectamine 2000 Transfection Reagent	Thermo Fisher Scientific	Cat# 11668019
Purelink HiPure Plasmid Miniprep Kit	Thermo Fisher Scientific	Cat# K210003
Purelink HiPure Plasmid Midiprep Kit	Thermo Fisher Scientific	Cat# K210005
Purelink HiPure Plasmid Maxiprep Kit	Thermo Fisher Scientific	Cat# K210007
Purelink HiPure Plasmid Gigaprep Kit	Thermo Fisher Scientific	Cat# K210009XP
Phusion High-Fidelity PCR Master Mix	New England Biolabs	Cat# M0531
Western Lightning Plus-ECL HRP Substrate	Revvity	Cat# NEL104001EA
Deposited data		
Cryo-EM map and Coordinates: ADAM17-Δ365iRhom1	This study	PDB: 9Q7Y
Cryo-EM map and Coordinates: ADAM17-Δ370iRhom1	This Study	PDB: 9XY4
Experimental models: Cell lines		
Expi293F cells	Thermo Fisher Scientific	Cat# A14527
U2 OS cells	ATCC	RRID: CVCL_0042
U2 OS iRhom1/iRhom2-KO Cells	This Study	N/A
Sf9 cells (*Spodoptera frugiperda* ovarian cells)	Expression Systems	Cat# 94-001F
Recombinant DNA		
mVenus-C1	Addgene	Plasmid # 27793
mVenus-C1-iRhom1	This Study	N/A
mVenus-C1-iRhom2	Maciag et al., 2025^39^	N/A
pFUSE-CHIg-hG1	InvivoGen	Cat# pfuse-hchg1
pFUSE2-CLIg-hk	InvivoGen	Cat# pfuse-hclk
pAPtag5-TGFα	Maciag et al., 2025^39^	N/A
pAPtag5-Epiregulin	This Study	N/A
pEG-iRhom1 plasmid	This Study	N/A
pEG-ADAM17 E406A plasmid	Maciag et al., 2025^39^	N/A
Software and algorithms		
FlowJo v10	BD Biosciences	https://www.flowjo.com
cryoSPARC	Structura Biotechnology	https://www.cryosparc.com
ChimeraX	UCSF	https://www.rbvi.ucsf.edu/chimerax
Topaz	Bepler et al.	https://github.com/tbepler/topaz
Coot	Emsley et al.	https://www2.mrc-lmb.cam.ac.uk/personal/pemsley/coot

## Highlights

- Cryo-EM structure of iRhom1 bound to the ADAM17 zymogen at near-atomic resolution
- Cytoplasmic domains of iRhom1 and iRhom2 regulate post-translational ADAM17 activation
- iRhom structural elements form a signaling relay across the membrane to activate ADAM17
