# Supplementary material for: Structural basis for ADAM17 activation by the iRhom1 pseudoprotease
Source: Cell Rep. Author manuscript; Available in PMC 2026 Jun 18. (PMC13277793; doi:10.1016/j.celrep.2026.117309)
Supplement: 1 [file NIHMS2181001-supplement-1.pdf]

Cell Reports, Volume *45*

Supplemental information

Structural basis for ADAM17 activation

by the iRhom1 pseudoprotease

Joseph N. Ungvary, Joseph J. Maciag, Hala F. Alnajjar, Eliud Morales Dávila, Conner E. Slone, Joel E. Thomas, Maria F. Rich, Sophia A. Carazo, Igal Ifergan, Jose Manuel Perez-Aguilar, Carl P. Blobel, and Tom C.M. Seegar

Supplemental Table 1. Cryo-EM Data Collection Parameters and Refinement Statistics.

**Protein**	zA17-(Δ365) iRhom1	zA17-(Δ370) iRhom1
RCSB: PDB ID	9Q7Y	9XY4
**Data Collection and Processing**		
Magnification	130 kX	105 kX
Voltage (kV)	300	300
Total Dose (e-/Å^2)	63.5	56.2
Defocus Range (μm)	0.8 - 2.2	0.4 - 2.2
Pixel Size (Å)	0.647	0.82
Symmetry	C1	C1
Initial Particle images	5.616×10^6	1.516×10^6
Final Particle Images	63,282	54,189
Map Resolution (Å)	3.37	3.17
Map Resolution Range (Å)	1.92 - 32.99	1.75 - 41.89
FSC Threshold	0.143	0.143
**Refinement**		
Model Composition		
Non-hydrogen atoms	10,530	10,223
Protein Residues	1,315	1,279
Ligands	10	10
B-factors (Å^2)		
Protein	126.86	37.85
Ligands	179.14	76.6
RMS deviations		
Bond lengths (Å)	0.006	0.005
Bond Angles (∘)	0.931	0.558
Validation		
MolProbity Score	1.88	2.2
Clashscore	9.46	8.95
Rotamer Outliers (%)	0.35	3.37
Ramachandran Plot		
Favored (%)	94.41	95.51
Allowed (%)	5.59	4.49
Disallowed (%)	0	0

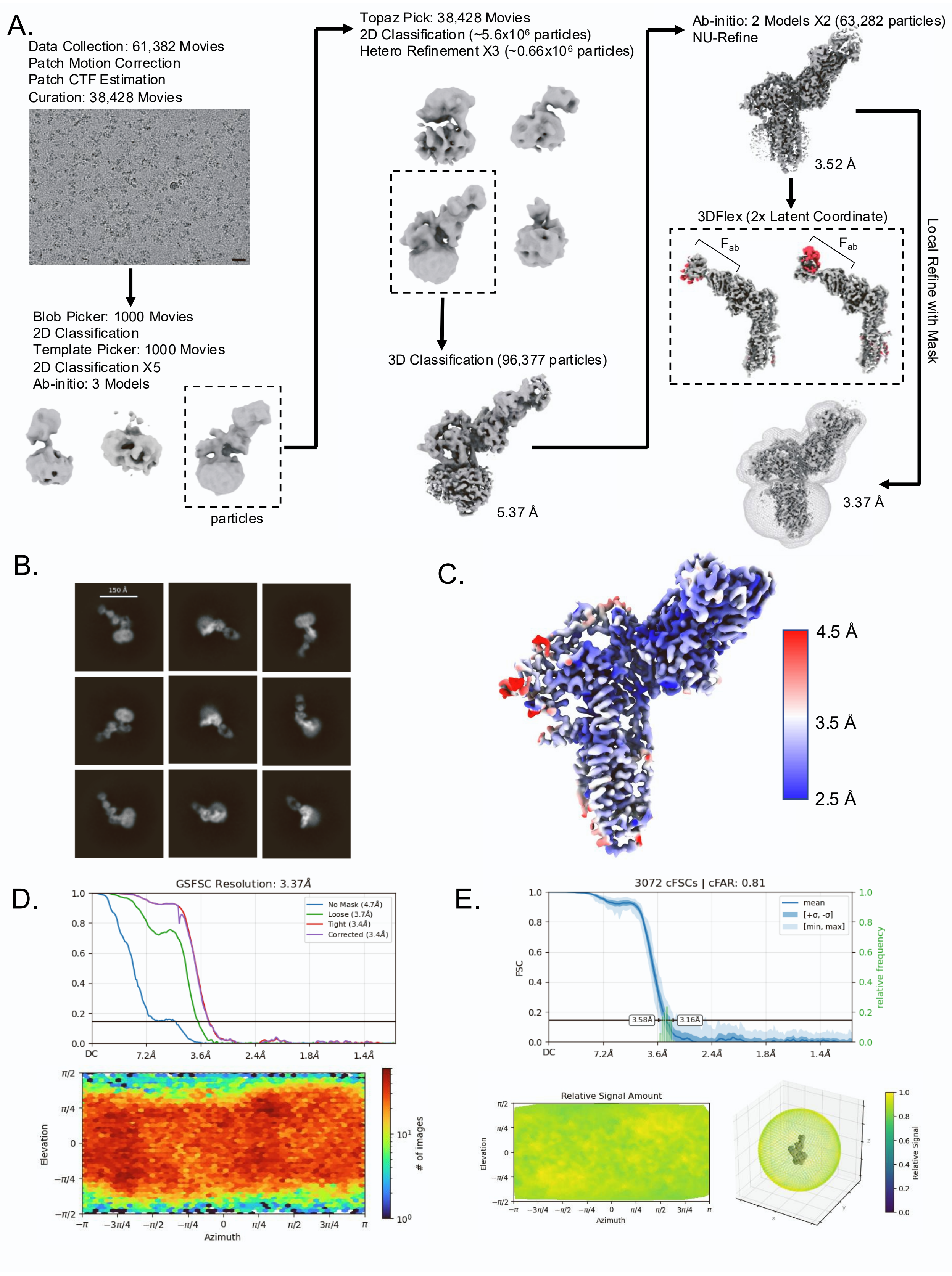

A.
Data Collection: 61,382 Movies
Patch Motion Correction
Patch CTF Estimation
Curation: 38,428 Movies
Blob Picker: 1000 Movies
2D Classification
Template Picker: 1000 Movies
2D Classification X5
Ab-initio: 3 Models
particles
Topaz Pick: 38,428 Movies
2D Classification (~5.6x10⁶ particles)
Hetero Refinement X3 (~0.66x10⁶ particles)
3D Classification (96,377 particles)
5.37 Å
Ab-initio: 2 Models X2 (63,282 particles)
NU-Refine
3.52 Å
3DFlex (2x Latent Coordinate)
Fab
Fab
Local Refine with Mask
3.37 Å
B.
150 Å
C.
4.5 Å
3.5 Å
2.5 Å
D.
GSFSC Resolution: 3.37Å
No Mask (4.7Å)
Loose (3.7Å)
Tight (3.4Å)
Corrected (3.4Å)
DC
7.2Å
3.6Å
2.4Å
1.8Å
1.4Å
Elevation
Azimuth
# of images
E.
3072 cFSCs | cFAR: 0.81
mean
[+σ, -σ]
[min, max]
FSC
relative frequency
3.58Å
3.16Å
Relative Signal Amount
Elevation
Azimuth
Relative Signal

Supplemental Figure 1. Workflow for structural determination of the ∆365-iRhom1-ADAM17-MEDI3622 F_{ab} Complex, related to Figure 1. (A) CryoSPARC processing workflow used to determine the zymogen ADAM17-∆365-iRhom1-MEDI3622 F_{ab} complex, with particle numbers indicated in parentheses and global resolutions at each step. Motion of the MEDI36221 F_{ab} constant domain is visualized from the 3DFlex maps at plus one (grey) and the difference map at minus one (red) standard deviations in the latent space. Micrograph scale bar is black at 300 Å in length **(B)** Representative 2D classes of the final particle subset with 150 Å scale bar. **(C)** Local resolution map of the final cryo-EM reconstruction. **(D)** Gold-standard Fourier shell correlation (FSC) curve (Top) used to estimate the overall resolution of the final cryo-EM map; the black line indicates the FCS value of 0.143. Angular distribution (Bottom) of the zymogen ADAM17-∆365-iRhom1-MEDI3622 F_{ab} particles used in the final cryo-EM map. (**E**) Orientation Diagnostic results showing the directional FSC (Top) and angular distribution (Bottom) analyses of particles contributing to the final cryo-EM reconstruction.

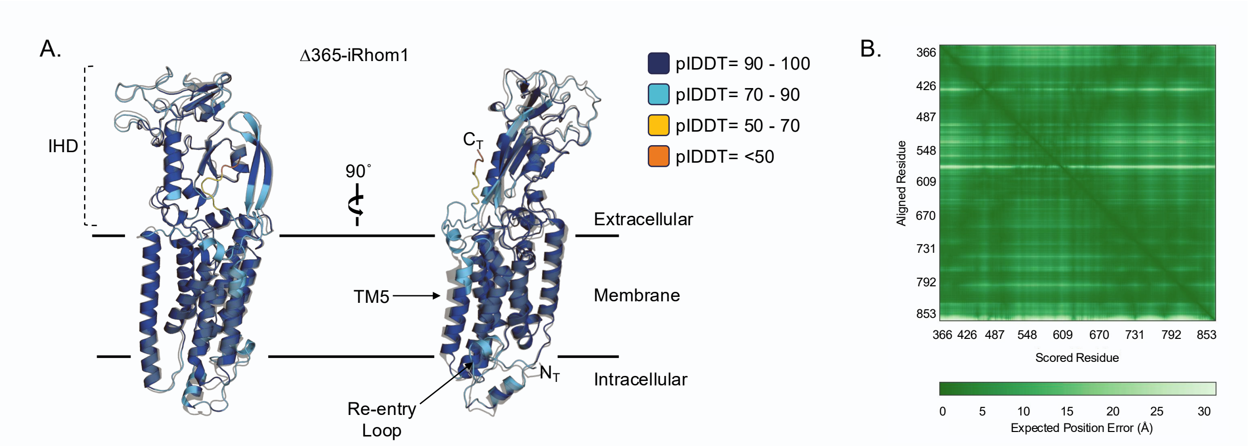

Supplemental Figure 2: Comparison of the predictive AlphaFold iRhom1 model to the cryo-EM iRhom1 structure, related to Figure 1. (A) Superimposition of the Δ365-iRhom1 AlphaFold model, shown as a cartoon and colored by pre-residue pLDDT confidence score, onto the Δ365-iRhom1 cryo-EM structure (grey). The approximate boundaries of the cell membrane are indicated with parallel black lines and key structural regions are annotated. **(B)** Predicted aligned error matrix plot for the Δ365-iRhom1 AlphaFold model.

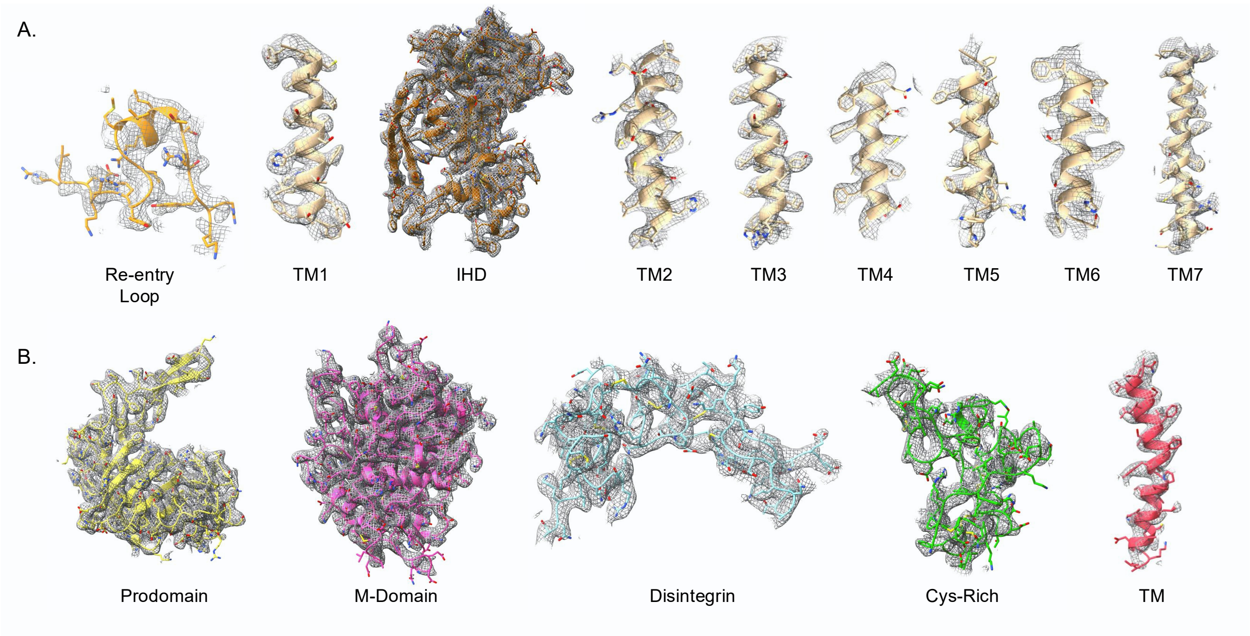

Supplemental Figure 3: Correlation of the final ADAM17 bound to Δ365-iRhom1 model fit into the cryo-EM map, related to Figure 1. Key structural features of (A) iRhom1 and (B) zymogen ADAM17 are shown as cartoon representations with side chain residues displayed as sticks and fitted into the B-factor sharpened cryo-EM map. Domains are labeled and colored as depicted in Figure 1A.

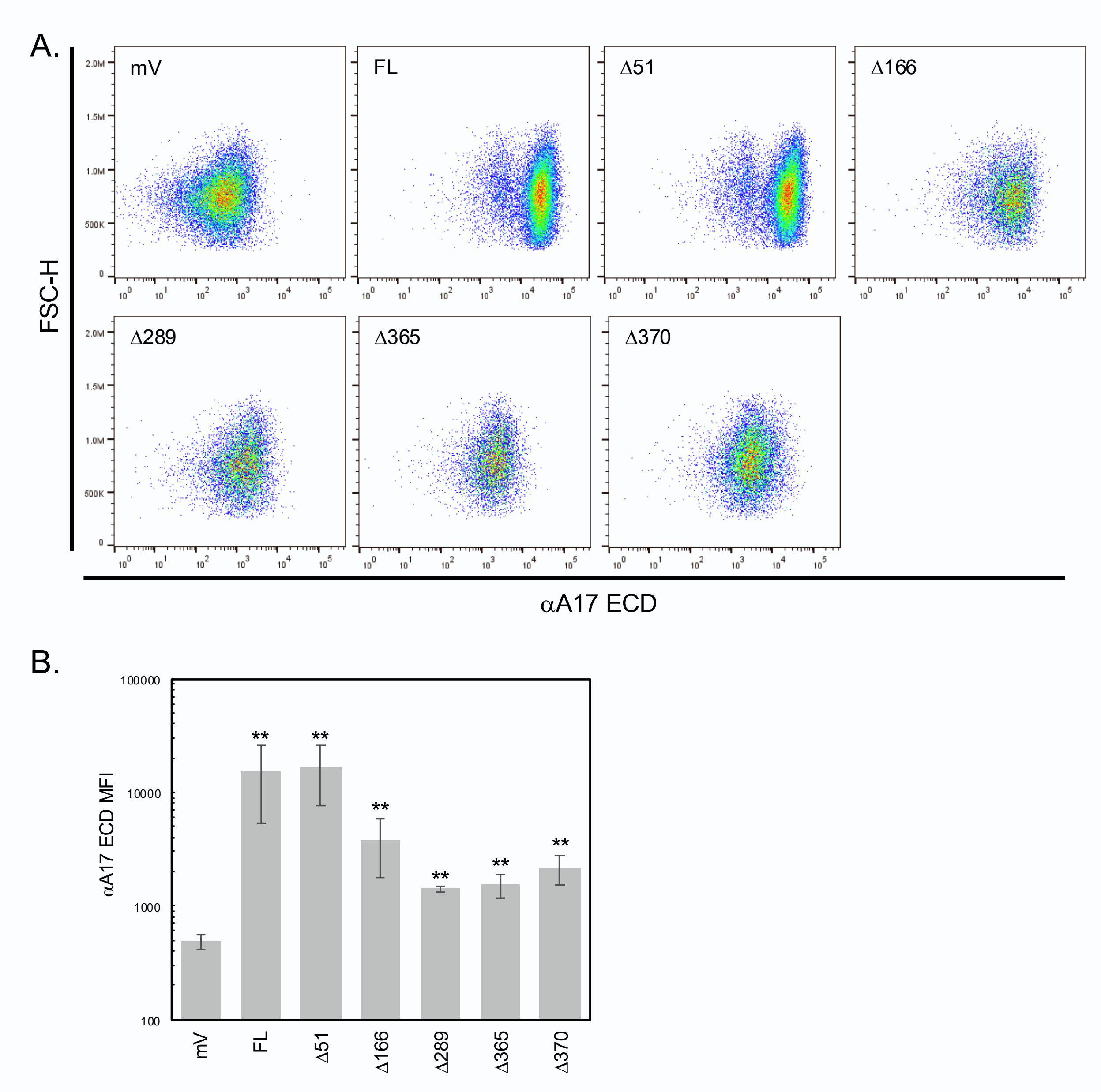

Supplemental Figure 4. Flow cytometry analysis of cell surface ADAM17 in iRhom1/2-null cells expressing mVenus-iRhom1 cytoplasmic truncations, related to Figure 2. (A) Scatter plots of ADAM17 surface levels detected with an αADAM17-ECD antibody in iRhom1/2 double null cells transfected with the different mVenus-iRhom1 cytoplasmic deletions. To eliminate non-transfected cells, quadrant gates were established based on the mVenus fluorescence signal and mVenus transfected cells

served as the negative control for ADAM17 staining. **(B)** Graphical representation of αADAM17-ECD mean fluorescence intensity (MFI) for each iRhom1 cytoplasmic truncation included. Statistical significance was determined on the log_{10} transformed MFI values using an unpaired, two-tailed t-test (where * represents $p < 0.05$; ** represents $p < 0.005$; NS represents not significant). Data are represented as the mean ± SD of N=3 independent experiments.

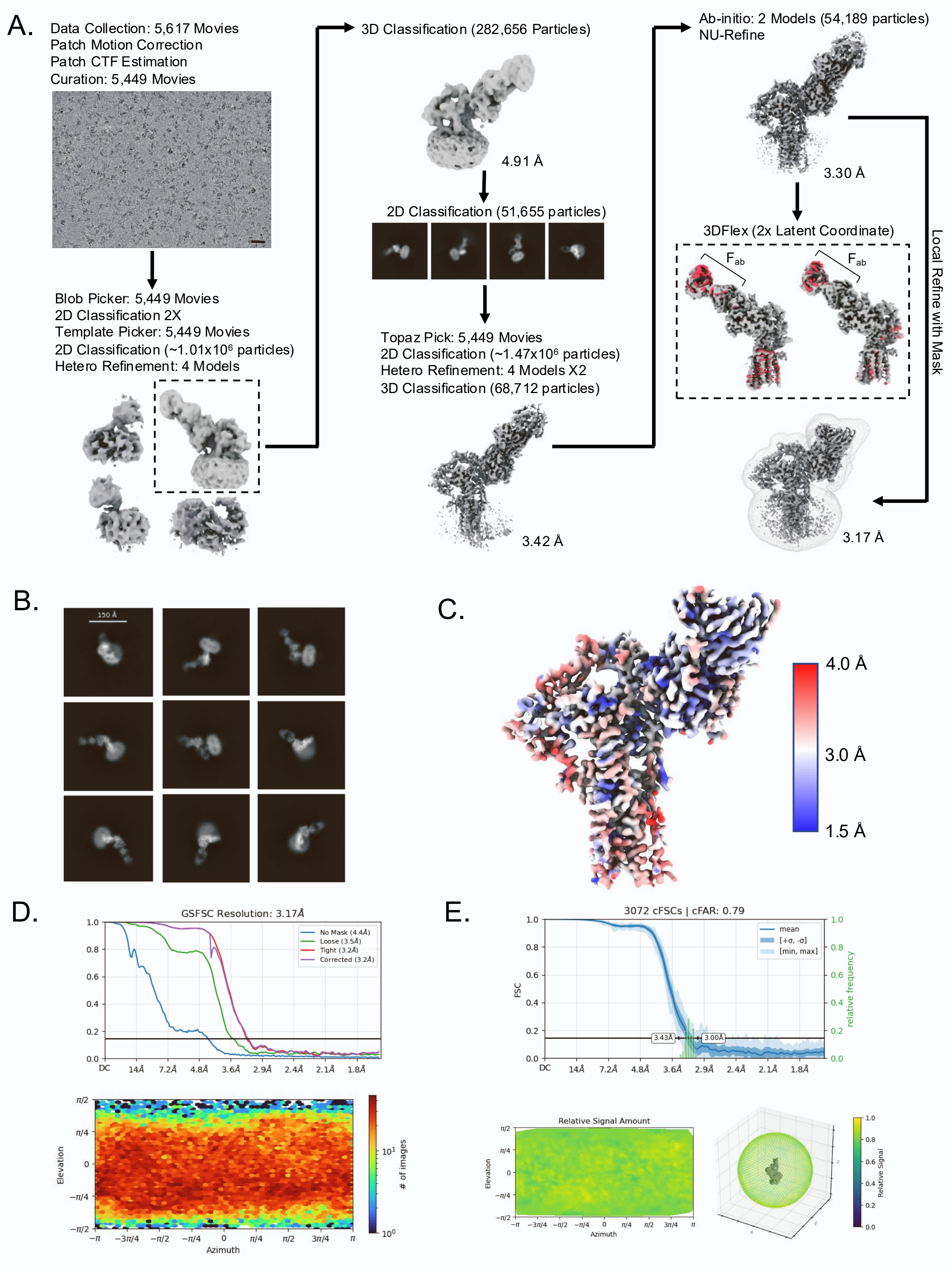
A.
Data Collection: 5,617 Movies
Patch Motion Correction
Patch CTF Estimation
Curation: 5,449 Movies
Blob Picker: 5,449 Movies
2D Classification 2X
Template Picker: 5,449 Movies
2D Classification (~1.01x10^6 particles)
Hetero Refinement: 4 Models
3D Classification (282,656 Particles)
4.91 Å
2D Classification (51,655 particles)
Topaz Pick: 5,449 Movies
2D Classification (~1.47x10^6 particles)
Hetero Refinement: 4 Models X2
3D Classification (68,712 particles)
3.42 Å
Ab-initio: 2 Models (54,189 particles)
NU-Refine
3.30 Å
3DFlex (2x Latent Coordinate)
Fab
Fab
Local Refine with Mask
3.17 Å
B.
150 Å
C.
4.0 Å
3.0 Å
1.5 Å
D.
GSFSC Resolution: 3.17Å
No Mask (4.4Å)
Loose (3.5Å)
Tight (3.2Å)
Corrected (3.2Å)
DC 14Å 7.2Å 4.8Å 3.6Å 2.9Å 2.4Å 2.1Å 1.8Å
Elevation
Azimuth
# of images
E.
3072 cFSCs | cFAR: 0.79
mean
[+σ, -σ]
[min, max]
FSC
relative frequency
3.43Å
3.00Å
DC 14Å 7.2Å 4.8Å 3.6Å 2.9Å 2.4Å 2.1Å 1.8Å
Relative Signal Amount
Elevation
Azimuth
Relative Signal

Supplemental Figure 5. Workflow of the structural determination of the zymogen Δ370-iRhom1-ADAM17-MEDI3622 F_{ab} Complex, related to Figure 4. (A) Workflow diagram containing the process used in CryoSPARC to determine the structure of the zymogen ADAM17-Δ370-iRhom1-MEDI3622 F_{ab} complex. Particle numbers used are indicated in parentheses. Motion of the MEDI36221 F_{ab} constant domain is visualized from the 3DFlex maps at plus one (grey) and the difference map at minus one (red) standard deviations in the latent space. Micrograph scale bar is black at 300 Å in length **(B)** Representative 2D classes of the final particle subset with 150 Å scale bar. **(C)** 3D model after last local refinement. Final resolution: 3.17 Å**. (D)** Gold-standard FSC (Top) used to determine overall resolution of the final cryo-EM map. The black line indicates the resolution corresponding to an FCS value of 0.143. Angular distribution (Bottom) of the zymogen ADAM17-Δ370-iRhom1-MEDI3622 F_{ab} particles used in the final cryo-EM map. (**E**) Orientation Diagnostic results showing the directional FSC (Top) and angular distribution (Bottom) analyses of particles contributing to the final cryo-EM reconstruction.

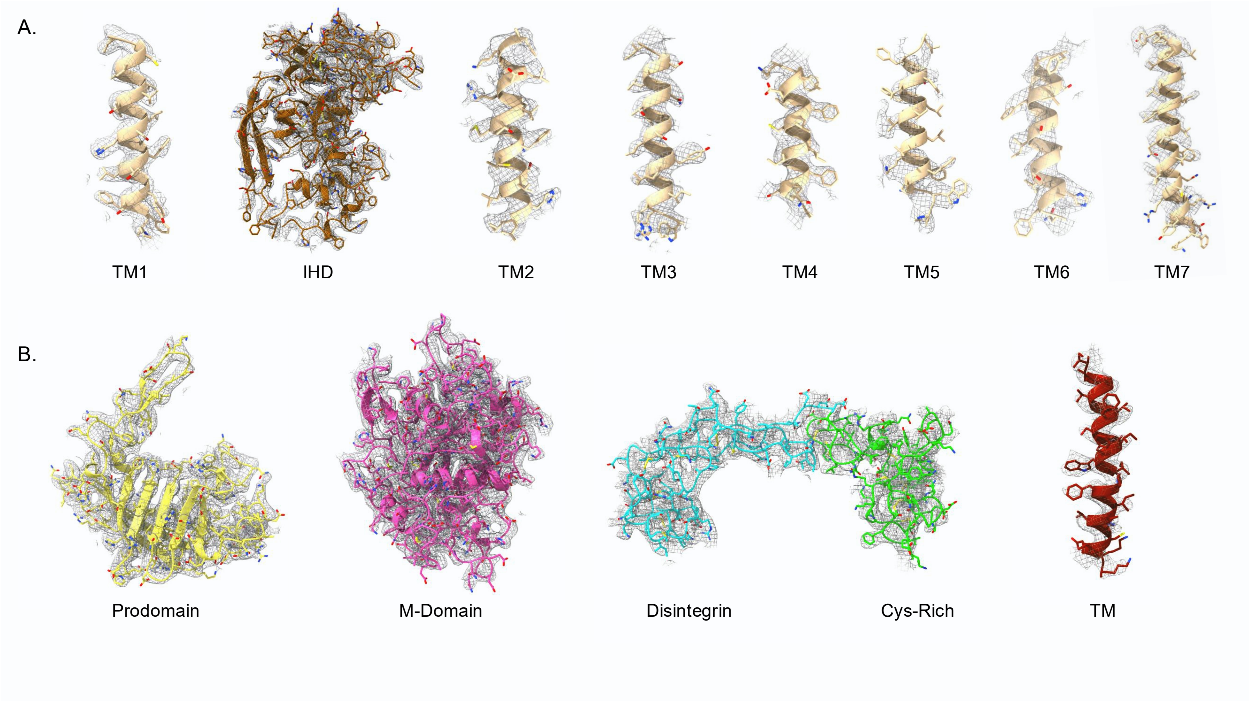

Supplemental Figure 6: Correlation of the final ADAM17 bound to Δ370-iRhom1 model fit into the cryo-EM map, related to Figure 4. Key structural features of **(A)** iRhom1 and **(B)** zymogen ADAM17 are shown as cartoon representations with side chain residues displayed as sticks and fitted into the B-factor sharpened cryo-EM map. Domains are labeled and colored as depicted in Figure 1A.

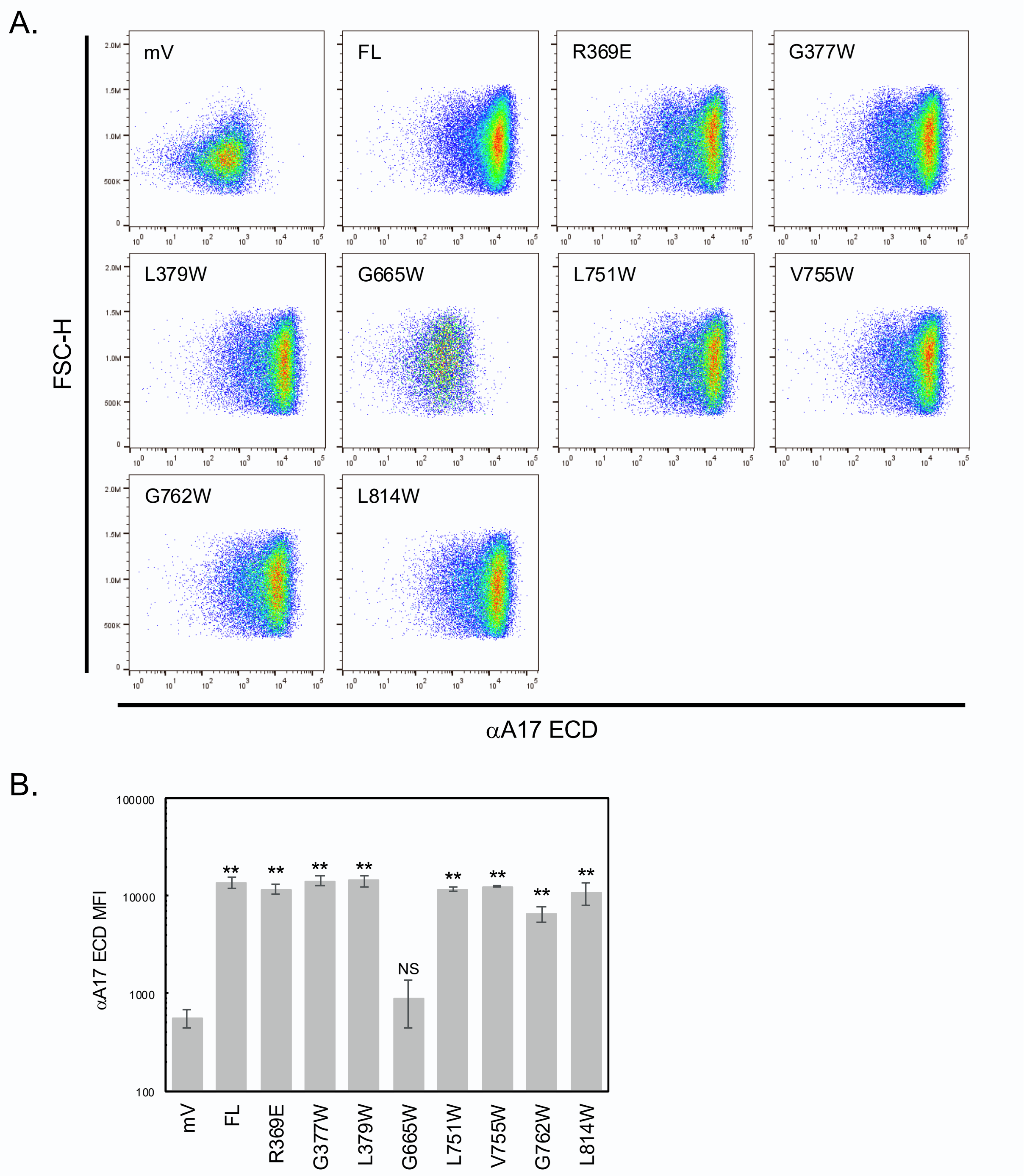

Supplemental Figure 7. Flow cytometry analysis of cell surface ADAM17 in iRhom1 site-directed mutations, related to Figure 5. (A) Scatter plots of ADAM17 surface levels detected with an αADAM17-ECD antibody in iRhom1/2 double null cells transfected with

the different mVenus-iRhom1 point mutations. Gates were established so that αADAM17-ECD staining was assessed from the mVenus-positive population of cells. **(B)** Graphical representation of αADAM17-ECD mean fluorescence intensity (MFI) for each iRhom1 site-directed mutant included. Statistical significance was determined on the $\log_{10}$ transformed MFI values using an unpaired, two-tailed t-test (where * represents $p < 0.05$; ** represents $p < 0.005$; NS represents not significant). Data are represented as the mean ± SD of N=3 independent experiments.

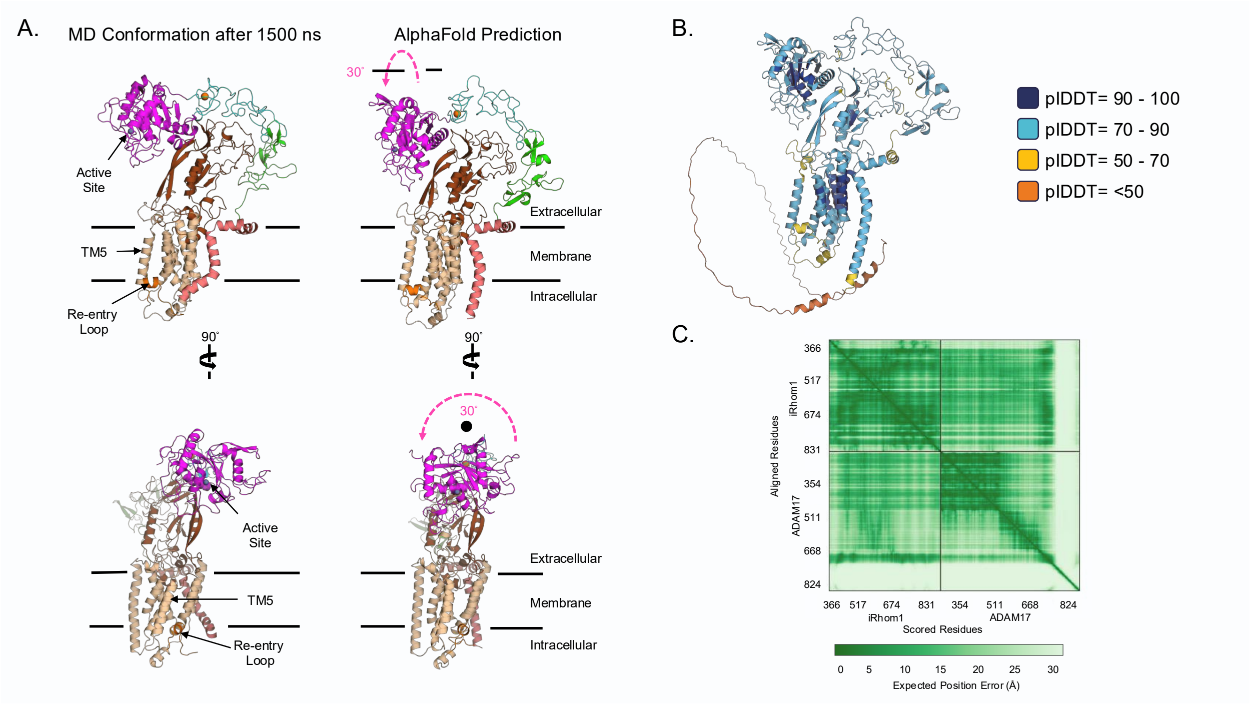

Supplemental Figure 8. Comparison of the predicted structure of the mature ADAM17 - iRhom1 complex generated by Molecular Dynamics versus AlphaFold, related to Figure 7. (A) Cartoon representation of the mature ADAM17-iRhom1 complex conformation after 1500 ns (left, see also Figure 6) compared to the AlphaFold predicted protein structure (right). The two structures readily superimpose with an overall RMSD of 1.9 Å. The primary difference between the two structures is a 30° rotation of the M domain along an axis parallel to the cell surface in the AlphaFold versus MD model (indicated in the bottom panel with the magenta arrow). The active site Zn ion (grey sphere), TM5 and re-entry loop are all labelled with arrows. **(B)** The mature ADADM17 and Δ365-iRhom1 AlphaFold model, shown as a cartoon and colored by pre-residue pLDDT confidence score. **(C)** Predicted aligned error matrix plot for the AlphaFold model of Δ365-iRhom1 bound to the mature ADAM17.

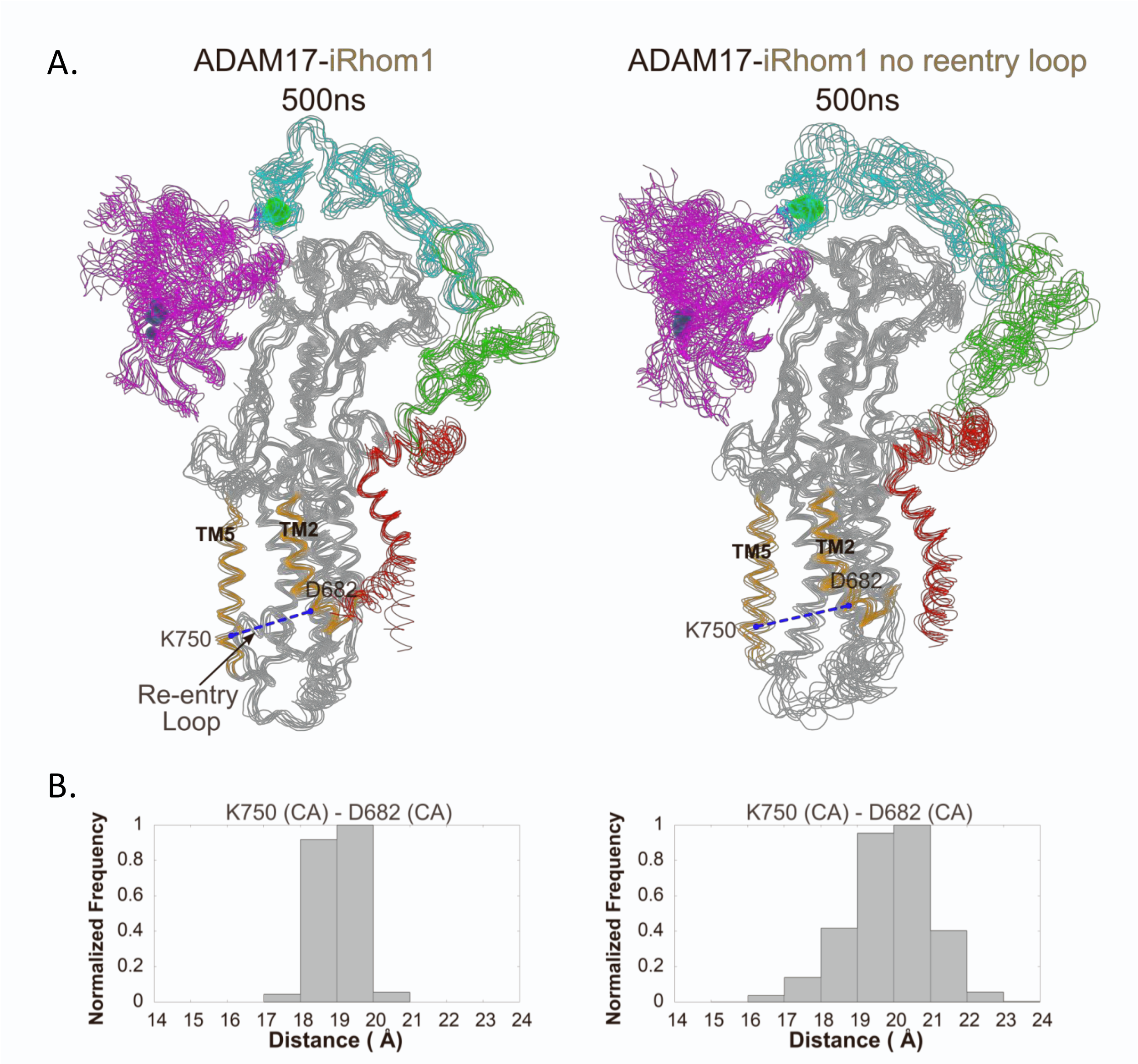

Supplemental Figure 9. MD Simulation of the AF-Derived mature ADAM17-iRhom1 complex without the Re-entry Loop, related to Figure 7. (A) Ribbon representation of the AF-derived mature ADAM17-iRhom1 (left) and Δre-entry loop (right) superimposed to different time intervals over the 500 ns of MD simulation. **(B)** Distribution of the distance between the α-carbon amino acid pair in the TM2 (D682) and TM5 (K750) connected by

blue dashed line in (A), plotted as frequency over the distance (Å) during the 500 ns MD simulation.
